# Towards verifying and improving estimations of China's CO_2_ and CH_4_ budgets using atmospheric inversions

**DOI:** 10.1093/nsr/nwaf090

**Published:** 2025-03-08

**Authors:** Yilong Wang, Yuzhong Zhang, Xiangjun Tian, Xuhui Wang, Wenping Yuan, Jinzhi Ding, Fei Jiang, Zhe Jin, Weimin Ju, Ruosi Liang, Xiao Lu, Lu Shen, Shuai Sun, Tao Wang, Hongqin Zhang, Min Zhao, Shilong Piao

**Affiliations:** State Key Laboratory of Tibetan Plateau Earth System, Environment and Resources (TPESER), Institute of Tibetan Plateau Research, Chinese Academy of Sciences, Beijing 100101, China; Key Laboratory of Coastal Environment and Resources of Zhejiang Province, School of Engineering, Westlake University, Hangzhou 310030, China; Institute of Advanced Technology, Westlake Institute for Advanced Study, Hangzhou 310030, China; State Key Laboratory of Tibetan Plateau Earth System, Environment and Resources (TPESER), Institute of Tibetan Plateau Research, Chinese Academy of Sciences, Beijing 100101, China; University of Chinese Academy of Sciences, Beijing 101408, China; Institute of Carbon Neutrality, Sino-French Institute for Earth System Science, College of Urban and Environmental Sciences, Peking University, Beijing 100871, China; Institute of Carbon Neutrality, Sino-French Institute for Earth System Science, College of Urban and Environmental Sciences, Peking University, Beijing 100871, China; State Key Laboratory of Tibetan Plateau Earth System, Environment and Resources (TPESER), Institute of Tibetan Plateau Research, Chinese Academy of Sciences, Beijing 100101, China; Frontiers Science Center for Critical Earth Material Cycling, Nanjing University, Nanjing 210023, China; Jiangsu Provincial Key Laboratory of Geographic Information Science and Technology, International Institute for Earth System Science, Nanjing University, Nanjing 210023, China; Institute of Carbon Neutrality, Sino-French Institute for Earth System Science, College of Urban and Environmental Sciences, Peking University, Beijing 100871, China; Frontiers Science Center for Critical Earth Material Cycling, Nanjing University, Nanjing 210023, China; Jiangsu Provincial Key Laboratory of Geographic Information Science and Technology, International Institute for Earth System Science, Nanjing University, Nanjing 210023, China; Key Laboratory of Coastal Environment and Resources of Zhejiang Province, School of Engineering, Westlake University, Hangzhou 310030, China; School of Atmospheric Sciences, Sun Yat-sen University, Zhuhai 519082, China; Guangdong Provincial Observation and Research Station for Climate Environment and Air Quality Change in the Pearl River Estuary, Key Laboratory of Tropical Atmosphere-Ocean System, Ministry of Education, Southern Marine Science and Engineering Guangdong Laboratory (Zhuhai), Zhuhai 519082, China; Department of Atmospheric and Oceanic Sciences, School of Physics, Peking University, Beijing 100871, China; Key Laboratory of Coastal Environment and Resources of Zhejiang Province, School of Engineering, Westlake University, Hangzhou 310030, China; State Key Laboratory of Tibetan Plateau Earth System, Environment and Resources (TPESER), Institute of Tibetan Plateau Research, Chinese Academy of Sciences, Beijing 100101, China; Institute of Atmospheric Physics, Chinese Academy of Sciences, Beijing 100029, China; State Key Laboratory of Tibetan Plateau Earth System, Environment and Resources (TPESER), Institute of Tibetan Plateau Research, Chinese Academy of Sciences, Beijing 100101, China; Institute of Carbon Neutrality, Sino-French Institute for Earth System Science, College of Urban and Environmental Sciences, Peking University, Beijing 100871, China

**Keywords:** carbon dioxide, methane, greenhouse gas, atmospheric inversion, China

## Abstract

This paper reviews the application of atmospheric inversions for estimating national CO₂ and CH₄ fluxes with a focus on China. After describing the fundamental principles and methodologies of the technique, we synthesize recent progress in estimating China's CO₂ and CH₄ budgets through atmospheric inversion, and compare these estimates with national greenhouse gas (GHG) inventory (NGHGI) reports. The inverted estimates for China's total CO_2_ and CH_4_ emissions amount to 8.35 ± 1.39 Pg CO_2_ a^−1^ and 60.8 ± 5.9 Tg CH_4_ a^−1^, respectively, in the last decade, which are in general consistent with NGHGIs. However, large uncertainties in spatial and temporal disaggregation of national budgets hinder the effectiveness of the method in verifying China's GHG budgets and improving NGHGI estimates. These uncertainties are largely driven by differences in inversion models, observational coverage and methodological assumptions. We recommend improving observational networks, conducting model intercomparison exercises and refining inversion methods to better support China's GHG reporting and future climate goals.

## Teaser

This article reviews the application of atmospheric inversions in verifying China’s CO_2_ and CH_4_ budgets, highlighting key challenges and offering recommendations for further improvement.

## INTRODUCTION

There is a broad scientific consensus that the Earth has been experiencing rapid warming at an unprecedented rate since the Industrial Revolution, and this warming is mainly driven by human-produced greenhouse gases (GHGs). To mitigate the risks and impacts of anthropogenic climate change, the Paris Agreement sets the long-term goal of keeping ‘the increase in the global average temperature to well below 2°C above pre-industrial levels’ and striving to limit the increase to 1.5°C [1]. Each Party to the Agreement has committed to preparing and submitting nationally determined contributions (NDCs) of GHG emission reductions, in accordance with the enhanced transparency framework (ETF). The global stocktake (GST) periodically assesses the collective climate progress towards the temperature goal of the Paris Agreement, mainly based on the national GHG inventories (NGHGIs) of emissions and removals submitted by each party to the United Nations Framework Convention on Climate Change (UNFCCC).

UNFCCC Annex I parties, including industrialized countries in the Organisation for Economic Co-operation and Development (OECD) at the time of 1992, and countries with economies in transition, are required to report their annual GHG inventories every year. On the other hand, non-Annex I parties, mostly developing countries and including China, are required to submit simplified reports in the form of National Communications (NCs), Biennial Update Reports (BURs) and Biennial Transparency Reports (BTRs). These reports are primarily estimations based on the ‘bottom-up’ approaches following the IPCC guidelines for NGHGI [2], where emissions are calculated by multiplying activity data with emissions factors and carbon removals are estimated based on carbon stock changes. However, these estimates have considerable uncertainties and do not account for fluxes from unmanaged lands. To support the assessment of NGHGI quality, the Intergovernmental Panel on Climate Change (IPCC) guidelines encourage verification activity through comparisons with independent flux estimates or with atmospheric measurements.

Verification of NGHGI with atmospheric measurements involves applying atmospheric transport models and atmospheric measurements of GHG mole fractions in an inverse analysis for estimating GHG fluxes. This approach is commonly referred to as the top-down method. Over the past decades, atmospheric inverse modeling has rapidly evolved into an important tool to evaluate bottom-up emission inventories. It is valued for its independence, objectiveness and global consistency, offering additional insights into the understanding of carbon cycles and climate change. Although not mandatory in IPCC guidelines, a number of countries (e.g. Australia, India, New Zealand, Switzerland, the UK and the USA) have incorporated atmospheric verification approaches in their NGHGI reports (https://unfccc.int/ghg-inventories-annex-i-parties/2023) and some other countries or regions are in the process of developing operational systems to support their NGHGI reports [3]. Beyond its role as a verification tool for NGHGI, inverse modeling itself can provide timely updates on emissions with only a few weeks’ or months’ delay, compared to the over-year-long lag for UNFCCC reports. This capability was demonstrated in the quick assessments of GHG from the COVID lockdowns [4] and the Nord Stream pipeline blowout [5].

China, currently the world's largest GHG emitter, has been regularly updating its NGHGIs. The latest submissions include its fourth NC [6] and third BUR [7] in 2023 and first BTR in 2024 [8]. These reports follow the requirements of the UNFCCC reporting guidelines for non-Annex I parties, and have taken into account the modalities, procedures and guidelines for the enhanced transparency framework under the Paris Agreement. Note that the first BUR in 2012 used the revised 1996 IPCC guidelines, while the other years follow the 2006 IPCC guidelines, introducing a difference in the land-use, land-use change and forestry (LULUCF) sector (discussed in the section **Inversion estimates of China's CO_2_ and CH_4_ fluxes—CO_2_**). China's BUR-3 report is developed in a bottom-up approach, including 51 key categories. The activity data were mainly from the continuous forest resources inventories for the LULUCF sector, and from the National Bureau of Statistics and other relevant departments for the other sectors. Wherever possible, higher-tier methods than IPCC Tier 1, along with country-specific emission factors, were used in these reports. However, China's NGHGI reports have not included verification through atmospheric inversion. The comparisons between the inversion and bottom-up estimates of China's GHG emissions were discussed in several papers [9–11], but these comparisons have been limited to only a few inversion systems, hindering a comprehensive understanding of the differences between the two approaches. With growing attention on China's GHG emissions, especially in light of its ambitious ‘carbon neutrality’ target, a wide-ranging collaboration from various institutions and agencies has compiled a comprehensive data set of China's GHG emissions in a recent synthesis [12]. In the meantime, China has submitted its third BUR, which estimates GHG emissions for 2018, providing an opportunity for a more focused investigation into the status of inversion estimates of China's GHG budgets.

This review summarizes recent advances in estimating China's CO_2_ and CH_4_ budgets based on atmospheric mole fraction measurements. We analyze the inversion results collected from the Global Carbon Project (GCP) for CO_2_ and multiple inversions from the literature for CH_4_. The knowledge gained from these inversions is valuable for guiding the Chinese and international groups in organizing inter-comparison exercises to better understand the uncertainties and limitations in current inversions and improve the robustness of atmospheric inversions in supporting China's future NCs and BURs.

## PRINCIPLES AND COMPONENTS OF INVERSE MODELING

### Inverse modeling principles

**Figure 1.**
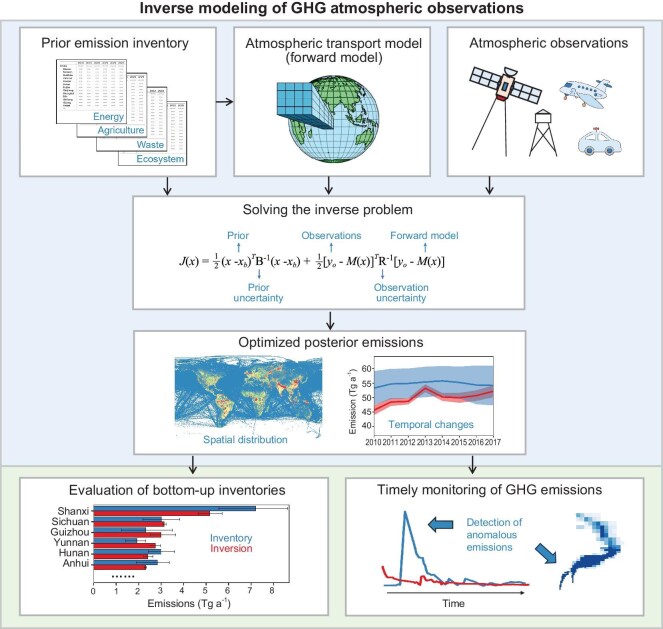
Framework of atmospheric inversions of GHG fluxes.

Atmospheric inversions for estimating CO_2_ and CH_4_ sources and sinks typically employ the statistical method based on conditional probability and the Bayes theorem [13,14]. In such an approach, a set of parameters gathered in a control vector (***x***; also known as a state vector), e.g. gridded fluxes to be estimated, are updated from prior estimates (***x***_b_) by incorporating the information from the atmospheric chemistry transport model and the measurements, aiming to minimize a cost function in the least square sense (Fig. 1):


(1) \begin{eqnarray*}
J\left(\boldsymbol x \right) &=& \frac{1}{2}{\left( {\boldsymbol x - {\boldsymbol x_b}} \right)^T}{{\bf{B}}^{ - 1}}\left( {\boldsymbol x - {\boldsymbol x_b}} \right)\\
&& +\, \frac{1}{2}{\mathrm{\,\,}}{[{\boldsymbol y_o} - M\left( \boldsymbol x \right)]^T}{{\bf{R}}^{ - 1}}{\mathrm{\,\,}}\left[ {{\boldsymbol y_o} - M\left( \boldsymbol x \right)} \right],\\
\end{eqnarray*}

where ***y***_o_ represents the set of measurements and *M*(***x***) is the model describing the relationship between the ***x*** space and the measurement space ***y*; B** and **R** are the covariance matrices of the prior error and observation error. The observation error encompasses transport model errors, measurement errors and any other sources of errors that affect the model-measurement misfits and are not controlled by the inversion [15], such as the representativeness error due to the resolution mismatch between model simulations and measurements [16] and the aggregation error when the inversion solves for fluxes of broad regions with heterogeneous flux error statistics [17].

The practical procedures adopted to minimize the cost function depend on the size of the matrices, the numeric operations involved, available computing resources and the linearity of the model *M*(***x***). In the case that *M*(x) can be expressed as a matrix operator **M**, the optimal estimate of the control variables, called the posterior estimate (***x***_a_) given the measurements and prior information, can be obtained:


(2) \begin{eqnarray*}
{\boldsymbol x_a} &=& {\boldsymbol x_b} + {\bf{B}}{{\bf{M}}^{\mathrm{T}}}{\left( {{\bf{MB}}{{\bf{M}}^{\mathrm{T}}} + {\bf{R}}} \right)^{ - 1}}\left( {{\boldsymbol y_o} - {\bf{M}}{\boldsymbol x_b}} \right)\\
&=& {\boldsymbol x_b}\! +\! {\left( {{{\bf{B}}^{ - 1}}\! +\! {{\bf{M}}^{\mathrm{T}}}{{\bf{R}}^{ - 1}}{\bf{M}}} \right)^{ - 1}}{{\bf{M}}^{\mathrm{T}}}{{\bf{R}}^{ - 1}}\left( {{\boldsymbol y_o}\! -\! {\bf{M}}{\boldsymbol x_b}} \right)\!,\\
\end{eqnarray*}

with its uncertainty characterized by the posterior error covariance matrix **A**:


(3) \begin{eqnarray*}
{\bf{A}} = {\left( {{{\bf{B}}^{ - 1}} + {{\bf{M}}^{\mathrm{T}}}{{\bf{R}}^{ - 1}}{\bf{M}}} \right)^{ - 1}}.
\end{eqnarray*}

Apart from this analytical solution [18–20], alternative methods are used when **M** cannot be explicitly stored in memory due to limited computing resources or when *M*(x) is highly non-linear. For example, the ensemble Kalman filter is a widely used algorithm [21–23] that can handle a very large number of variables without storing full error covariance matrices, updating the fluxes sequentially in discretized time windows. The variational approach [24–26] applies to cases where the matrices are too large to store in memory or the explicit computation of **M** is computationally expensive. This method involves iteratively evaluating the gradient of the cost function and seeking the descent direction toward the minimum. While the variational approach can be applied across many areas of atmospheric sciences, it often requires specialized programming.

The inverse problem of estimating CO_2_ and CH_4_ fluxes is usually underdetermined, given the fact that fluxes are ubiquitous over almost all the Earth's surface while the measurements are relatively sparse (see section **Principles and components of inverse modeling—Atmospheric measurements**). Therefore, the first term of Equation (1) regulates the solution of the inverse problem, preventing it from deviating unrealistically far from the prior estimates (see section **Principles and components of inverse modeling—Control variables and their prior information**).

The uncertainties in estimated fluxes are essential to understanding the difference between top-down and bottom-up approaches. There are generally two classes of methods to characterize the uncertainties in estimated fluxes, the Bayesian posterior uncertainty given by Equation (3) and the uncertainty across an ensemble of different inversions. The Bayesian posterior uncertainty quantifies how the observation and prior errors impact the posterior flux estimates. The ensemble-based method incorporates a range of uncertainties related to inversion configurations such as transport models, inversion algorithms, choices of prior fluxes and error statistics. This method has been widely adopted by intercomparison activities including the GCP [27,28], OCO-2 modeling intercomparison project (MIP) [29] and European atmospheric transport inversion comparison (EUROCOM) project [30]. These two methods explore different aspects of inversion uncertainties and are in many cases complementary to each other.

### Atmospheric measurements

The measurements of atmospheric mole fractions of GHG are a key component of atmospheric inversions. The measurements can be grouped into two types: ground-based measurements and satellite-based remote sensing. Ground-based measurements typically refer to those made near the Earth's surface, ranging from a few meters to several kilometers above the ground, such as those collected on towers or onboard ships, aircraft or balloons. These measurements can be obtained through discrete sampling, such as using flasks, or through continuous observations with *in-situ* instruments. Atmospheric gas measurements are typically reported as dry-air mole fractions (mol mol^−1^) or, equivalently, as volume mixing ratios, commonly represented in units like parts per million (ppm). The longest records of contemporary atmospheric CO_2_ mole fractions started in the 1950s, at La Jolla Pier (California), Mauna Loa (Hawaii) and the South Pole Observatory (https://www.scrippsco2.ucsd.edu/data/atmospheric_co2/). Since then, the number of GHG stations has greatly increased, and there are more than 200 stations across the globe (https://gaw.kishou.go.jp/), but they are disproportionally located in developed countries. In China, the longest-running atmospheric observations of GHGs started in 1982 at Shangdianzi. In the last decade, atmospheric measurements of GHG have been expanded to more than 100 stations over China, mainly operated by China's Meteorology Agency (CMA) [31].

Remote sensing techniques retrieve GHG mole fractions by measuring spectrally resolved radiations at specific wavelengths that are sensitive to the variations of GHG mole fractions in the atmosphere. These retrievals are usually expressed in terms of column-averaged dry-air mole fractions, XCO_2_ and XCH_4_. Among some of the most important operating satellites for CO_2_ monitoring are the Japanese Greenhouse gases Observing SATellite (GOSAT) and GOSAT-2, NASA's second and third Orbiting Carbon Observatory (OCO-2 and OCO-3), and China's TanSat and Atmospheric Environmental Monitoring Satellite (AEMS). In addition to CO_2_, the GOSAT series has the ability to measure methane (CH_4_), together with the TROPOspheric Monitoring Instrument (TROPOMI) onboard Sentinel-5 Precursor, forming the most widely used public platforms for high-precision CH_4_ monitoring from global to regional scales, and even down to point sources. In addition, hyperspectral and multi-spectral spectrometers, such as GHGSat, MethaneSAT, Sentinel-2, PRISMA, GaoFen-5 and Ziyuan-1, with targeted observing capability, are also carrying out CH_4_ emission monitoring for large point sources. Although satellites generally offer a wide coverage of measurements over the globe, most of the abovementioned satellites, except AEMS, are equipped with passive sensors, whose soundings are often obscured by thick clouds and aerosols. For example, satellite measurements have a lower frequency of obtaining high-quality retrievals in Southeast China during the monsoon period compared to the non-monsoon period. In addition, high-quality retrievals are also limited over complex topography such as the Tibetan Plateau, which hampers the robustness of inversion results over these regions (see section **Inversion estimates of China's CO_2_ and CH_4_ fluxes—CO_2_**).

### Atmospheric chemistry transport model

Inference of the distributions and magnitudes of GHG sources and sinks from measured variations in atmospheric mole fractions requires an atmospheric chemistry transport model describing the quantitative relationship between surface fluxes and atmospheric mole fractions, which constitute a key part of the *M*(***x***).

Different models vary in their level of detail and complexity, but they have to account for processes like wind-driven advection of GHG plumes, horizontal and vertical mixing, chemical reactions, and the enhancement or depletion of gases due to various sources and sinks. The representation of these processes in analytical, deterministic or stochastic ways leads to the Gaussian, Eulerian and Lagrangian models. The Gaussian (plume) model yielding a normally distributed mole fraction field is usually applied to point source assuming constant emissions and stationary wind. The Eulerian models discretize the atmospheric space into a set of grid boxes and simulate the transport between these boxes (e.g. GEOS-Chem [32], TM5 [33], LMDz [34] models). The Eulerian models are widely used in both regional and global studies. The Lagrangian models simulate the motion of air masses by an ensemble of virtual particles statistically following a given wind field (e.g. STILT [35], HYSPLIT [36], FLEXPART [37]). The Lagrangian model is commonly used for regional applications.

One of the key aspects of transport models is the model resolution, which impacts the advection, as well as horizontal diffusion over heterogeneous topography and underlying surface and the vertical mixing within and above the planetary boundary layer, which may in turn impact the inversion results. Typical resolution of transport models has evolved in the past decades, from typically 10° × 10° with 10 vertical layers [38,39] down to 1–2° horizontal and 20 to 80 vertical layers [40,41] globally. Some models with nesting or zooming ability, as well as regional models, can downscale the resolution to a few kilometers for specific regions of interest [42,43].

### Control variables and their prior information

The control variables are designed to consider the physical and chemical processes that link the fluxes and atmospheric signals. In the inversion of CO_2_ fluxes, fossil fuel and cement CO_2_ emissions are usually assumed to be well-known and not optimized, and only the fluxes from the biosphere and ocean are solved. In the inversion of CH_4_ fluxes, some inversions solve for the total CH_4_ fluxes, while some other inversions separately solve for fluxes from different source categories with distinct spatial and temporal variations. Unlike CO_2_ inversions, anthropogenic fluxes are also optimized in CH_4_ inversions due to large uncertainties in anthropogenic fluxes relative to natural fluxes.

The temporal and spatial discretization of the fluxes to be controlled is at the discretion of each inversion. Some inversions solve for fluxes at large scales, for example, for a few large regions and with annual to monthly intervals. This strategy easily fits the main policy demand and has a historical background due to limited computing resources, but introduces the so-called ‘aggregation error’ [17]. Recent inversions have largely improved the resolution to small regions [18] or even at the highest resolution of the transport model [40].

The prior information of the fluxes is usually obtained from various flux products, such as inventories of anthropogenic emissions, process-based models for natural fluxes, remote sensing flux estimates and statistical models. The choices of prior flux estimates differ among inversion systems. Prior flux fields can affect the inverted fluxes [44], especially over poorly sampled regions, necessitating improvements in prior flux estimates. However, there is no consensus on which flux product is superior to the others, and therefore a wide range of flux products are used as priors in different inversion systems, even in intercomparison projects like GCP, OCO-2 MIP and EUROCOM.

## COMPARISON BETWEEN INVERSION ESTIMATES AND NGHGI

In this study, we collect a set of inversions of China's CO_2_ and CH_4_ fluxes to compare with China's NCs and BURs at the policy-relevant national and annual scales. We extract China's GHG fluxes from gridded flux products using an exclusive economic zone mask [45], which includes offshore CH_4_ emissions from oil and gas production. In addition, we also compare the spatial and temporal patterns of the inverted fluxes across different inversions. Because the CO_2_ flux estimates from inversions and NGHGI differ in terms of definition and included processes, some adjustments to the inversion results are necessary to ensure comparability with NGHGI reports. In the following, we describe the inversions used and the adjustments made.

For the CO_2_ budget, 14 inversions from the Global Carbon Budget report version 2023 and three inversions from the China Greenhouse Gas emission data set (CNGHG) version 2023 [12] are collected. While the Global Carbon Assimilation System (GCASv2) and Global ObservatioN-based system for monitoring Greenhouse GAses (GONGGA) inversions are included in both data sets, we only keep those in CNGHG to avoid redundancy of the same inversions. Most inversions solved for the fluxes all over the globe and the fluxes over China are extracted. In addition, one regional inversion with a resolution of ∼50 km is included for China's CO_2_ fluxes. The details of the inversions are summarized in Table S1. The ground-based measurements assimilated in the inversions are mainly from the ObsPack GLOBALVIEWplus v8.0 and NRT v8.1 data sets, including eight stations in China, six of which only cover the period between 2010–2016. As a result, China's inverted CO_2_ fluxes from the eight ground-based inversions are mostly constrained by CO_2_ gradients from the sites upwind and downwind of China. The seven satellite-based inversions cover a shorter period than ground-based inversions due to the availability of satellite observations.

All these inversions solved for the net biosphere-atmosphere exchange CO_2_ fluxes (NBE), with emissions from energy and industrial processes sectors (usually termed as ‘fossil fuel and cement emissions’ in inversions [46]) kept unoptimized in the total CO_2_ budget. Considering the difference between NBE and stock change represented by NGHGI, we account for the effects of reduced C compounds (RCC) and lateral fluxes. RCC are the substances emitted mainly as CH_4_, carbon monoxide (CO) and biogenic volatile organic compounds (BVOCs) from both anthropogenic and natural sources. These compounds are oxidized to CO_2_ in the atmosphere in hours to days (BVOCs), months (CO) and ∼10 years (CH_4_) after their instantaneous emissions. Atmospheric CO_2_ mole fraction changes are not influenced by RCC emissions, but include the signals of RCC oxidization which are diffusively distributed over the globe. However, current inversions assume that all the C in combusted fossil fuel is emitted as CO_2_, leading to an overestimation of fossil fuel CO_2_ emissions. No current atmospheric inversions of CO_2_ fluxes have included these RCC loops, leading to an overestimation of land CO_2_ sink over regions with intensive RCC emissions [29]. Previous studies estimated that the RCC effect is the largest correction to be made [10,16] for China's CO_2_ budget. The lateral fluxes involve the carbon initially fixed as CO_2_ in one place and released in another place, including the carbon leached into rivers and then exported into the ocean, and the imports/exports of crop, wood and animal products. In this study, we used the adjustment values from a recent study [16], with lateral fluxes updated by Deng *et al.* [47], ranging from 0.063 to 0.085 Pg CO_2_ yr^−1^. Of note, China's NGHGI reports only account for fluxes over managed land, which is ∼80% of China's total land area [6], while inversions include all the land area. No gridded map is available in China's NGHGI reports to differentiate between managed and unmanaged land. But the unmanaged land was primarily located in western China [48] where fluxes are very small. As a result, the definition of managed land in this comparison has a negligible impact (Fig. S1). We compare the adjusted inversion NBE with the sum of the carbon budgets from the agriculture sector and the LULUCF sector from China's NGHGI reports. In addition, we also compare the total carbon budget (TCB) of China. That is the sum of adjusted NBE and fossil fuel and cement emissions from inversions, and the sum of emissions from energy, industrial processes, agriculture, LULUCF and waste sectors from NGHGI reports.

For CH_4_ budget, we collect 20 inversions from the Global Methane Budget report version 2020 [28], two inversions from CNGHG [12] and 14 published studies from the literature (Table S2). These inversions differ in their study periods, observations (ground-based, satellite and combined), transport models (Eulerian, Lagrangian and hybrid) and inversion set-ups (including prior estimates and error statistics) and therefore reflect the spectrum of inversion uncertainties. Based on national total and anthropogenic methane fluxes reported from these inversions, we evaluate the uncertainties of inversions and compare the inversion results with NGHGI. The NGHGI accounts for all major anthropogenic methane sources, i.e. fossil fuel, agriculture and waste and LULUCF sectors, but does not include natural methane sources like natural wetlands, freshwater bodies, geological seeps and wildfires. Within the NGHGI framework, the LULUCF sector specifically addresses methane emissions from human-managed or altered wetlands. However, many inversion studies classify wetlands as natural sources, thereby excluding wetland emissions from anthropogenic fluxes. Hence, we compare anthropogenic fluxes from inversion studies with NGHGI values without LULUCF. Detailed information on the location and extent of managed or altered wetlands in NGHGI, which is currently unavailable, will be useful for refining the assessment.

Moreover, inversion studies applied different approaches to separate anthropogenic and natural fluxes. Some studies subtracted bottom-up natural fluxes from the inverted total flux [48], some made the attribution based on prior fractions of natural fluxes in individual grid cells [18], and some optimized different sectors separately in the inversion [49]. Using studies reporting both total and anthropogenic fluxes, we also evaluate the uncertainty arising from the partition between anthropogenic and natural fluxes.

## INVERSION ESTIMATES OF CHINA'S CO_2_ and CH_4_ FLUXES

### CO_2_

**Figure 2.**
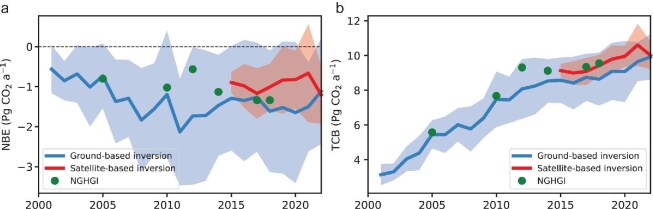
China's (a) net biosphere-atmosphere exchange (NBE) and (b) total carbon budget (TCB) estimates from inversions and national GHG inventories (NGHGIs). Negative values represent carbon sinks that remove CO_2_ from the atmosphere, and positive values represent carbon sources that release CO_2_ into the atmosphere. The lines represent mean values of the ensemble of inversions, and the shaded area represents the 1-σ uncertainty envelope.

Figure 2 shows the time series of China's annual NBE and TCB estimates from inversions and NGHGIs. Note that the NGHGI of 2012 used the revised 1996 IPCC guidelines, which only consider the fluxes from forest area in the LULUCF sector and have a less negative value (smaller carbon sink) than other years, while the other years follow the 2006 IPCC guidelines and include fluxes from forest land, cropland, grassland, wetlands, settlements and other land. The NBE estimates from ground-based inversions are well consistent with the NGHGIs, except for 2012 due to the different land area represented. The satellite-based inversions cover a shorter period and are also consistent with the NGHGI for the recent two years within the uncertainty. In addition, both inversions also match the NGHGIs in TCB within their uncertainties, but satellite-based inversions show a slightly closer match with NGHGIs and a smaller spread than ground-based inversions. Note that the fossil fuel and cement CO_2_ emissions used in inversions are slightly smaller than the NGHGIs in 2017 and 2018 (Fig. S2), but are not optimized due to the current sparse density of the observations. This bias can propagate into NBE estimates, e.g. a slight underestimation of NBE in 2018 in satellite inversions. This underscores the need for future development in observational networks and inversions to better separate CO_2_ fluxes of different origins (see section **Prospects and recommendations**).

Although the ensemble mean of inversions shows a general consistency with NGHGIs, there are significant uncertainties among different inversion estimates. The uncertainty range of satellite-based inversions is ∼60% smaller than ground-based inversions for both NBE and TCB estimates. This might be due to the wider coverage of measurements from satellites than the limited number of ground-based stations in China [29,50], indicating the benefit of satellite measurements and the necessity to improve the ground-based network (see section **Prospects and recommendations—Observations**).

The standard deviation of annual NBE during the 2015 to 2018 period obtained in each inversion is on average 56% of the standard deviation across all inversions for a given year, indicating that different inversions may have systematic offsets. There is a low consistency in the interannual variability from different inversions, indicated by the mean correlation in the NBE time series among inversions being 0.16. Furthermore, the distinct inter-annual variabilities (IAVs) between ground-based and satellite-based inversions reflect the differences in the observations, as exemplified by the same inversion system assimilating those observations (Fig. S3), challenging the rigorous detection of interannual variability and the drivers from the current ensemble of inversions. The relatively flat trends in NGHGIs (except 2012) compared to inversions further adds to this difficulty. Although a previous study [51] compared ground-based and satellite-based inversion results with environmental conditions during 2015–2019 and suggested better constraints from satellite observations than from the limited coverage of ground-based observations to capture the impact of extreme drought over China, the distinct IAVs between ground-based and satellite-based inversions, particularly for the year 2022, underscores the importance of a long-term and continuous evaluation of inversion results, with independent evidence (see section **Prospects and recommendations—Evaluation of atmospheric inversions**).

All the inversions and NGHGIs consistently indicate that China's terrestrial ecosystem acts as a carbon sink, but the spatial distribution of this sink varies across inversions (Fig. S4). For example, CarboScope and Nonhydrostatic Icosahedral Atmospheric Model-based Inverse Simulation for Monitoring CO_2_ (NISMON) display a dipole pattern with sharply contrasting strong sources and sinks, while others do not. The two Coperncus Atmosphere Monitoring Service (CAMS) inversions assimilating ground-based and satellite observations exhibit a similar spatial distribution. Likewise, the two GONGGA inversions, one global and one regional system, also show comparable spatial patterns. This similarity may relate to the use of the same inversion algorithm, prior fluxes and/or the transport model within variants of a given system, highlighting the need for dedicated sensitivity experiments to test the impacts of such differences (see section **Prospects and recommendations—Inversion intercomparison and development**).

### CH_4_

**Figure 3.**
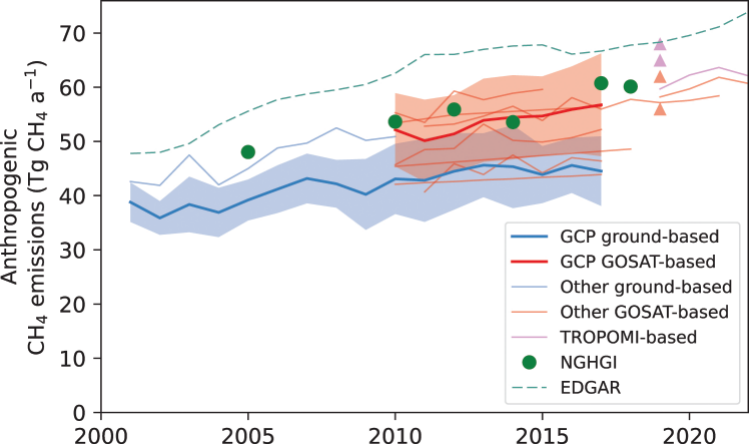
China's anthropogenic CH_4_ emissions from inversions, NGHGIs and Emissions Database for Global Atmospheric Research (EDGAR). The thick lines represent mean values of the GCP ensemble of inversions, and the shaded area represents the 1-σ uncertainty envelope. The light lines and triangles represent estimates derived from individual inversions other than the GCP ones.

Figure 3 shows the estimates of China's CH_4_ anthropogenic emissions from the ensemble of inversions compiled in this study. The annual mean total fluxes from China during 2010–2018 are in the range of 46–63 Tg a^−1^ (Fig. S5) with the contribution from anthropogenic sources between 38–62 Tg a^−1^ (Fig. 3). The NGHGI values, whether inclusive or exclusive of LULUCF, fall within the range of anthropogenic emissions reported by satellite-based inversions studies for 2010–2018, but out of the upper 1-σ range of the ground-based inversions (Fig. 3). The ground-based inversion estimates are significantly lower than GOSAT-based estimates and NGHGI values (*p* < 0.05). This discrepancy may be attributed to the limited number of surface stations in China, with most of these stations being background stations located far from areas with significant anthropogenic emissions. In addition, our ensemble includes several inversions of TROPOMI data, which have become available since 2018. Results from TROPOMI-based inversions are generally higher than GOSAT-based results of the same year (Fig. 3), which has been found partly attributable to biases in TROPOMI retrievals [52,53]. Further investigations are needed to identify any systematic biases between ground-based, GOSAT and TROPOMI observations, so that these observations can be used to derive consistent inversion results.

Besides NGHGI, we also compare the inversions with the Emissions Database for Global Atmospheric Research (EDGAR) bottom-up inventory, which has been widely used by inversion studies as prior information. China's anthropogenic CH_4_ emissions inferred by inversions are consistently lower than the estimate by EDGAR v8.0, which averages 66 Tg a^−1^ for 2010–2018 and 71 Tg a^−1^ for 2019–2022. Consequently, this comparison suggests that EDGAR likely overestimates China's anthropogenic CH_4_ emissions, and NGHGI estimates are more consistent with independent atmospheric signals, illustrating the role of atmospheric inversions in verifying bottom-up estimates.

Most of the long-term inversions indicate increases in China's CH_4_ emissions over the past decade, which are in the range of 0.3–0.9 Tg a^−2^ around the 2010–2018 period (Fig. 3). In comparison, various bottom-up inventories in the literature do not converge on the sign of the recent trend after 2013 [54,55]. NGHGIs also show a general rise in emissions since 2005, with the largest increase occurring between 2014 and 2017. One of the 11-year inversions found that this trend slowed down after 2015, which is consistent with the recent compilation of CNGHG [12].

Compared with anthropogenic emissions, natural CH_4_ fluxes are relatively small in China, but their uncertainties affect the comparison between inversion results and NGHGI. Although the total budgets are constrained by atmospheric observations, the partitioning between anthropogenic and natural fluxes is contingent on the methodologies adopted by various inversions and often heavily relies on prior information. Based on inversions that report both anthropogenic and total emissions, we find that the fraction of anthropogenic fluxes in total emissions varies between 80% to 97%, while natural CH_4_ fluxes exhibit a 4-fold uncertainty, ranging from 1.9 to 8.2 Tg a^−1^ (Fig. S6).

**Figure 4.**
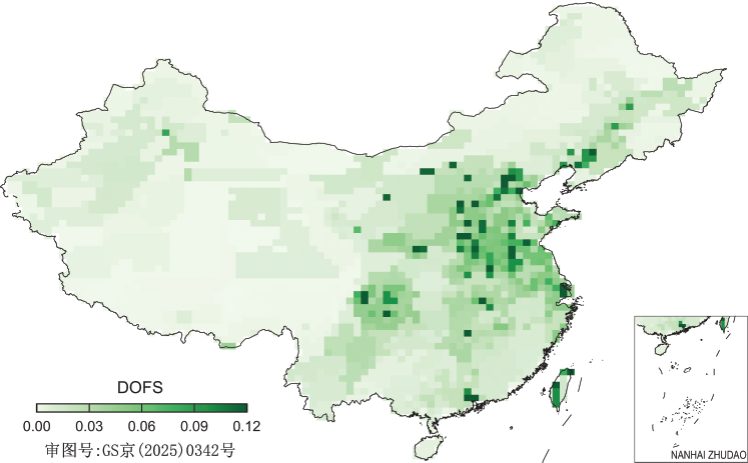
Averaging kernel sensitivities derived from an inversion of GOSAT CH_4_ observations for 2010–2017. A value of 1 indicates full constraints provided by observations and a value of 0 signifies no observational constraints.

In addition to national totals, inverse studies provide valuable insights into the spatial, temporal and sectoral distribution of China's CH_4_ emissions and their trends. Several studies have identified that the overestimation by EDGAR is primarily linked to emissions from coal mines in North China [56,57]. In terms of the emission changes, it was found that China's CH_4_ emissions exhibit spatially contrasting trends between 2010 and 2017, with increases in North China and decreases in Southwest China. These patterns are attributed to the geographic redistribution of coal production within the country [18]. The slowdown of China's CH_4_ emission growth after 2015 was largely due to emission reductions from wetlands and agriculture sectors [49]. Moreover, using satellite or ground-based observations, recent regional studies report inverse analyses over small but critical regions within China, providing in-depth understanding of CH_4_ emissions from specific cities (e.g. Hangzhou and Taiyuan; [58,59]) or sources (e.g. rice emissions in Northeast China; [60]). However, the ability of the inversions to provide spatial, temporal and sectoral information varies greatly with region and season, partly due to uneven data coverage across the country. For example, Fig. 4 illustrates the spatial distribution of averaging kernel sensitivities (diagonal elements of **I**-**AB**^−1^; see Equations 2 and 3) for a GOSAT inversion during 2010–2017 [18], which measures the amount of independent information provided by the observations. This figure shows that satellite observations have relatively good constraint over North and East China, but weaker constraints over Central and South China. This is mainly due to extensive cloud cover during summertime over Central and South China, which reduces coverage of satellite observations.

## PROSPECTS AND RECOMMENDATIONS

Encouraged by the IPCC guidelines for national GHG reporting, we reviewed the recent achievements in atmospheric inversions of CO_2_ and CH_4_ fluxes with a focus over China. The ensemble means of inverted CO_2_ and CH_4_ emissions from China amount to 8.35 ± 1.39 Pg CO_2_ a^−1^ and 60.8 ± 5.9 Tg CH_4_ a^−1^ in the last decade. We found that the inversion estimates are broadly consistent with NGHGI values within their large spreads. However, current inversions are prone to huge uncertainties, limiting their roles in verifying China's GHG budgets. Particularly, we found that the spreads of atmospheric inversions exceed 15% at the national scale (Figs 2 and 3) and even larger at subnational scales (Fig. S4), and that the IAVs from different inversions do not converge. In the following, we propose recommendations for the future development of atmospheric inversions for countries like China.

### Observations

One of the bottlenecks of robust inversion estimates of China's CO_2_ and CH_4_ budgets is the limited number of observations (Figs S7 and S8). For example, the averaging kernel sensitivities in Fig. 4 that are significantly below 1 over all grid cells illustrate that the independent constraints brought by GOSAT observations are very lacking. This lack of constraints is even exacerbated over Central and South China due to the extensive cloud cover during summertime. This limits the ability of existing satellite measurements to effectively capture and constrain, for instance, rice emissions—the major CH_4_ source in these regions during summer. Future refinements in inverted GHG budgets will depend on expanding observational data, standardized data calibration and improved validation from multiple observing platforms.

The first generation of CO_2_ and CH_4_ satellites (e.g. GOSAT, GOSAT-2, OCO-2, OCO-3 and TanSat) were designed to explore methodologies for measuring CO_2_ and CH_4_ from space with required qualities to estimate GHG fluxes. Planned future missions will expand and enhance current observing platforms to constitute operational systems for national GHG monitoring. These efforts will include missions such as MicroCarb, Sentinel-5, CO2M and China's follow-on TanSat mission, with broad-swath imaging capability. In addition, satellites dedicated to point source monitoring, mainly for CH_4_, are also joining and have improved the point source detection threshold from TROPOMI at 25 tons h^−1^ [61] down to 100 kg h^−1^ [62]. Active sensors, currently only AEMS and soon Methane Remote Sensing Lidar Mission (MERLIN), using lidars instead of reflected and scattered sunlight as light sources, could provide measurements during the nighttime and at high latitudes in the winter.

While expanding space-based observing platforms is beneficial for all countries and continents, ground-based measurements will remain critical for China. Ground-based measurements provide complementary observational information to satellites in regions where, and during periods when, dense clouds and aerosols obscure satellites’ view. Moreover, variations in ground-based mole fractions typically represent the signals within the planetary boundary layer where most GHG fluxes are occurring, while satellite column-integrated mole fractions combine air of various ages and thus flux signals from larger areas than the native retrieval footprints. Zhang *et al.* demonstrated that including surface observations from seven surface sites across China improves observational constraints, especially over East and Northeast China, where satellite measurements are sparse owing to frequent cloud and snow surface conditions [19]. Wang *et al.* proposed a network consisting of 60 ground-based stations and showed that an optimal design of the locations of these stations can potentially reduce the posterior uncertainty of the estimate of China's land carbon sink by 80% [63]. Similarly, surface site distributions aiming for improved monitoring efficiency of CH_4_ have also been explored [64].

Other atmospheric tracers can provide useful tools for further refining CO_2_ and CH_4_ fluxes, particularly their source attribution, which is stressed by IPCC guidelines in the accounting of national total emissions and targeted mitigation efforts. In traditional CO_2_ inversions, fossil fuel emissions are assumed with no uncertainty, but this is not valid at subnational scale and high-resolution grid boxes. Radiocarbon is a useful tracer to separate fossil fuel CO_2_ emissions and ecosystem fluxes [20,65]. Measurements of NO_2_, which is available through a ground-based air quality network and satellite remote sensing, can also be useful for quantifying fossil fuel CO_2_ emissions [4,66]. For CH_4_ inversion, measurements of ^13^C/^12^C ratios in atmospheric CH_4_ contain information for distinguishing different CH_4_ sources with pyrogenic, thermogenic and biogenic origins [67–69]. In addition, ethane is co-emitted with CH_4_ from fossil fuel sources [70], CO is widely used to detect the changes in biomass burning emissions [69], and ammonia is predominantly from agricultural emissions [71], all contributing to the range of measurements useful for GHG monitoring.

Further advancements in atmospheric inversions will be possible through combining all kinds of relevant observational constraints from multiple satellite and ground-based platforms. Actually, some abovementioned measurements have been routinely made by several official agencies or research institutions. For example, CMA's ground-based CO_2_ and CH_4_ observation network includes seven Global Atmosphere Watch (GAW) global/background stations [31] and more than 100 regional/provincial stations (https://english.www.gov.cn/news/202312/02/content_WS656a6af2c6d0868f4e8e1cfb.html). ^14^C measurements are made in 15 Chinese cities [72]. NO_2_ and CO is routinely monitored across China for air quality monitoring.

All the measurements made by different stations and institutions must be cross-calibrated against common standards, such as the World Meteorological Organization (WMO) GAW calibration scale, to ensure precision and trueness of measurements. This is essential to eliminate artificial gradients between stations before integrating the data into atmospheric inversion systems to estimate CO_2_ and CH_4_ fluxes. Such compatibility goals are routinely assessed by, for instance, the WMO and International Atomic Energy Agency (IAEA) ‘Round Robin’ Comparison Experiment and the European Integrated Carbon Observation System (ICOS) ‘labeling process’ [73]. This practice is, at least, not yet operational across different institutions in China.

In addition to standardized data calibration, public data sharing is vital for advancing GHG monitoring and inversion studies by fostering collaboration and promoting transparency. International platforms such as ICOS have demonstrated the value of openly accessible data from extensive observational networks, enabling researchers to validate models, improve inversion estimates and better understand GHG fluxes at regional and global scales. While significant progress has been made in expanding atmospheric measurement networks in China, the limited availability of publicly accessible data restricts broader scientific collaboration and integration into global inversion frameworks. Establishing a public data-sharing platform for China's observational networks would not only strengthen domestic research capabilities but also support global efforts in GHG monitoring and verification under the ETF. Such an initiative would complement ongoing efforts by various institutions to provide more accurate and reliable estimates of China's GHG budgets.

It is also essential to ensure compatibility between satellites and ground-based measurements. It has been reported that satellite observations may be subject to various types of biases and uncertainties in some regions, impacted by surface albedo and aerosol loadings [74,75]. However, current space-based measurements can only be evaluated against ground-based remote sensing retrievals from the Total Column Carbon Observing Network (TCCON) [76] and COllaborative Carbon Column Observing Network (COCOON) [77], while these ground-based remote sensing measurements, in turn, need to be compared with vertical profiles obtained by *in-situ* instruments to align with WMO/GAW standards. As of now, China has only two TCCON sites, in Heifei and Xianghe [78,79], and the footprint of the existing TCCON network has large gaps in coverage in other parts of China [80]. Without robust evaluation data, it was shown that TROPOMI observations of XCH_4_ tend to have high biases in East China and Northwest China compared to GOSAT [52]. While important, the vertical profile measurements are supported by short-term funding in China [81]. Continuous support of these activities will be crucial for improving satellite retrievals and thus top-down CO_2_ and CH_4_ flux estimates.

### Inversion intercomparison and development

In this review, a large set of the inversions are collected from GCP, which are designed for constraining budgets at the global scale. The inversion designs in GCP are not strictly adapted to national and subnational budget accounting, and allow for the participation of a wide range of inversion systems with various configurations. This strategy allows for the representation of the full range of uncertainty space, but the contributions of different drivers in the inversion uncertainty have yet to be explored. There is an urgent need to organize dedicated intercomparisons to assess the uncertainties in atmospheric inversions for China's GHG budgets. The knowledge gained from GCP and EUROCOM, along with the initial findings from the synthesis of China's GHG budget [12], can aid in the development of a rigorous protocol, involving a series of sensitivity experiments, that controls one driver at a time.

Firstly, the prior fluxes have a significant impact on the posterior estimates from inversions [55], depending on the strength of observation constraints that often vary spatially and temporally. Therefore, inversion results should be interpreted by accounting for the difference in prior inventories. In the GCP and EUROCOM ensembles, different inversions are allowed to choose their own prior fluxes, such that the ensemble spread includes the effect of prior uncertainty, but the contribution of different priors to the overall uncertainty is not quantified. In some scientific research, a small set of prior fluxes were used but a systematic assessment is lacking [18,49]. Moreover, recent studies proposed a framework that mathematically accounts for the influence of prior information. This framework facilitates accurate comparisons of inversions employing different prior information, as well as evaluations of inversions against various bottom-up emission inventories [82,83]. In the synthesis of China's GHG budget [12], detailed and harmonized bottom-up inventories are derived, which can serve as common baseline priors used by the intercomparison.

Secondly, biases in atmospheric transport models and associated meteorological inputs have been recognized as a major source of uncertainty in the inversion results. The influences are less pronounced at the global scale due to the mass conservation but become increasingly more significant from hemispheric to regional scales. For instance, studies have shown that inversions using coarse-resolution global GEOS-Chem simulations tend to overestimate China's terrestrial CO_2_ sink due to the underestimation of vertical mixing, compared to inversions using other transport models [84,85]. The EUROCOM inversion intercomparison assessed the inversion results over Europe using several mesoscale regional models and a global model. They concluded that, at least for inversions over Europe, the model resolution seems not a major limitation of global models, although high-resolution models can better resolve atmospheric processes and vertical mixing [86,87]. Future attempts to intercompare China's GHG flux inversions should aim to assess this error source by including inversion systems with a variety of transport models.

While uncertainty in atmospheric transport modeling is evident in both global and regional models, regional systems developed by local specialists enable detailed assessments of large-scale GHG fluxes across the country as well as localized emission hotspots. Regional inversions, in principle, can be well-suited to addressing country-specific priorities, such as verifying sectoral emissions or assessing the effectiveness of mitigation policies at scales ranging from the provincial to infrastructure level. However, such analyses require dense observational networks, as discussed above, to effectively constrain emissions and removals from heterogeneous flux fields. Additionally, the resolutions of regional systems may range from ∼50 km down to the kilometer scale to meet the demands of different applications within a limited domain. Accurate estimations of GHG fluxes in a regional inversion require unbiased boundary conditions, which are often challenging. Addressing this issue would necessitate integration with global systems or the advanced coupling of systems at different resolutions to ensure consistency in inverted fluxes across scales, from the kilometer scale to the national scale.

In the future development of inversions, we also recommend considering the source attribution in inversions. Specifically, in CO_2_ inversions, the separation between fossil fuel CO_2_ emissions and CO_2_ fluxes from terrestrial ecosystems is a priority of the IPCC guideline [2]. This can be achieved by assimilating the radiocarbon measurements in the inversions [20,65], and some studies have shown that existing satellites can potentially constrain emissions from large emitters like megacities and power plants under preferable weather conditions [88–90]. Future satellites with imaging capability are expected to become the cornerstone of such anthropogenic emission quantification [3,91]. Furthermore, a number of projects are also focusing on the development of inversion systems dedicated to separately estimate fossil fuel emissions and agricultural, forestry and other land use (AFOLU) fluxes, such as the CO_2_ Human Emissions (CHE) project (https://www.che-project.eu/), the Copernicus CO_2_ service (CoCO2) project (https://coco2-project.eu/), the VERIFY project (https://verify.lsce.ipsl.fr/) and the Pilot Application in Urban Landscapes towards integrated city observatories for greenhouse gases (PAUL) project (https://www.icos-cp.eu/projects/icos-cities). In terms of CH_4_ inversions, we suggest that future developments may include the capability to assimilate measurements of multiple species (e.g. ^13^CH_4_ and ethane) to enhance source attribution and to incorporate multiscale measurements (e.g. area measurements from TROPOMI and point source measurements from GF-5) to improve estimations for intensive sources like coal mining [92].

Improving the correction term to enable robust comparisons between inversion results and NGHGIs remains a non-trivial task. Over the long term, RCC emissions and lateral fluxes may exhibit trends that cannot be overlooked [47]. For example, the combustion efficiency of carbon-based fuels has been steadily improving over the past decades [93], likely reducing RCC emissions, which constitute the largest correction. To better address these fluxes, we recommend fostering broader collaboration with researchers specializing in these areas. Such interdisciplinary efforts would enhance the robustness of atmospheric inversions as a tool for verifying national GHG budgets.

In addition to CO_2_ and CH_4_, N_2_O emissions contribute significantly to China's GHG budget and are reported in China's NGHGIs. However, there have been only a limited number of N_2_O inversions. Preliminary attempts supported by the GCP indicated that the inverted anthropogenic N_2_O emissions, averaged from three inversions, closely matched China's NGHGI values [48]. Its recent update incorporating four inversions estimated emissions to be 36% higher [47]. This highlights that the limited number of available N_2_O inversions makes comparison with NGHGIs more challenging for N_2_O than for CO_2_ and CH_4_. Therefore, new N_2_O inversions are urgently needed to better verify China's N_2_O emissions.

### Evaluation of atmospheric inversions

The substantial spread of current inversion results highlights that the inverse problem for CO_2_ and CH_4_ fluxes, at least in the case of China, remains largely underdetermined. Critical to reducing the breadth of the uncertainty is systematic evaluation of the inversion performance, which typically requires comparing inversion results with independent observation data.

The evaluation can be done by comparing simulated mole fractions driven by posterior fluxes with mole fraction measurements that have not been assimilated in the inversion [21,25,94]. In GCP, the quality of participating inversions is routinely evaluated using aircraft measurements in the free troposphere along north-to-south transects, with a focus on the global budgets [27]. The assessment of regional results depends on the availability of suitable data. For example, independent vertical CO_2_ gradients were used to constrain inversion results, revealing a weaker carbon sink in the northern hemisphere than previous studies, and suggesting that the tropics are nearly carbon neutral [95,96]. A recent study combined the aircraft measurements over the tropical Atlantic Ocean and the suite of OCO-2 MIP inversions to develop the emergent constraints, falsifying the large carbon emission estimates by some satellite-based inversions [97]. These examples underscore the need for independent measurement data to assess the inversion results for China. However, such data are currently limited in China. We encourage joint efforts between research institutions and aviation/ship companies to initiate aircraft/ship campaigns over and downwind of China. Additionally, we propose exploring vehicles and trains as platforms to gather regional transect measurements, and aircraft and drones to obtain vertical profile observations. The horizontal and vertical gradients revealed in these observations are critical for evaluating inversed fluxes and model transport.

Evaluations have also been carried out using eddy-covariance flux measurements [98]. There are more than 200 eddy-covariance sites across China, which can serve as the pillar for evaluating inversion results. However, caution should also be taken when comparing inverted fluxes with these flux measurements due to the differences in spatial scales. Inversions typically represent fluxes over hundreds of kilometers, covering multiple ecosystems within a grid cell, while flux measurements generally represent fluxes over several kilometers, often capturing only one specific ecosystem.

Integrated evaluations of inverted fluxes, spanning national to kilometer scales, require comprehensive analysis of the strengths and weaknesses of each type of independent data mentioned above. This process will help identify gas in the current evaluation of GHG inversions and further guide the design of new independent observations with meticulous consideration of their locations and types.

## FINAL REMARKS

The CNGHG project brought together the scientific community and agencies responsible for China's NGHGIs, integrating both bottom-up and top-down approaches. As part of this activity, this paper synthesizes the current status and uncertainties in top-down estimates of China's CO_2_ and CH_4_ budgets. Drawing insights from this synthesis, we propose recommendations for enhancing observational data, conducting inversion intercomparisons, developing inversion methods and evaluating inversion performance to improve atmospheric inversions for China's GHG budgets. We envision that atmospheric inversions will serve as a powerful tool to cater to various requirements within the ETF, contributing to scientific dialogues on China's GHG emission changes and their underlying drivers, thereby advancing progress towards the nation's ambitious carbon neutral target.

## Supplementary Material

nwaf090_Supplemental_File

## Data Availability

The inverted CO_2_ fluxes from GCP version 2023 are publicly available at https://doi.org/10.18160/4M52-VCRU. The national anthropogenic CH_4_ emissions from inversions in the GCP are available at https://doi.org/10.5281/zenodo.5089799. The inversions of CO_2_ and CH_4_ fluxes from CNGHG are available at http://carbon.pku.edu.cn.
