# Supplementary material for: Towards verifying and improving estimations of China's CO2 and CH4 budgets using atmospheric inversions
Source: Natl Sci Rev. 2025 Mar 8;12(4):nwaf090. doi: 10.1093/nsr/nwaf090 (PMC12089751; doi:10.1093/nsr/nwaf090)
Supplement: nwaf090_Supplemental_File [file nwaf090_supplemental_file.pdf]

Supplemental Information for

Towards verifying and improving estimations of China's CO_2 and CH_4 budgets using atmospheric inversions

Yilong Wang[1], Yuzhong Zhang[2,3*], Xiangjun Tian[1,4], Xuhui Wang[5], Wenping Yuan[5], Jinzhi Ding[1], Fei Jiang[6,7], Zhe Jin[5], Weimin Ju[6,7], Ruosi Liang[2], Xiao Lu[8,9], Lu Shen[10], Shuai Sun[2], Tao Wang[1], Hongqin Zhang[11], Min Zhao[1], Shilong Piao[5]

[1] State Key Laboratory of Tibetan Plateau Earth System, Resources and Environment (TPESRE), Institute of Tibetan Plateau Research, Chinese Academy of Sciences, Beijing, 100101, China
[2] Key Laboratory of Coastal Environment and Resources of Zhejiang Province, School of Engineering, Westlake University, Hangzhou, 310030, China
[3] Institute of Advanced Technology, Westlake Institute for Advanced Study, Hangzhou, 310030, China
[4] University of Chinese Academy of Sciences, Beijing, 101408, China
[5] Institute of Carbon Neutrality, Sino-French Institute for Earth System Science, College of Urban and Environmental Sciences, Peking University, Beijing, 100871, China
[6] Frontiers Science Center for Critical Earth Material Cycling, Nanjing University, Nanjing, 210023, China
[7] Jiangsu Provincial Key Laboratory of Geographic Information Science and Technology, International Institute for Earth System Science, Nanjing University, Nanjing, 210023, China
[8] School of Atmospheric Sciences, Sun Yat-sen University, Zhuhai, 519082, China
[9] Guangdong Provincial Observation and Research Station for Climate Environment and Air Quality Change in the Pearl River Estuary, Key Laboratory of Tropical Atmosphere-Ocean System, Ministry of Education, Southern Marine Science and Engineering Guangdong Laboratory (Zhuhai), Zhuhai, 519082, China
[10] Department of Atmospheric and Oceanic Sciences, School of Physics, Peking University, Beijing 100871, China
[11] Institute of Atmospheric Physics, Chinese Academy of Sciences, Beijing, 100029, China

Correspondence to: Yuzhong Zhang (zhangyuzhong@westlake.edu.cn)

This PDF file includes:

Supplementary Tables S1 to S2

Supplementary Figures S1 to S8

Supplementary Tables

Table S1 Inversion-based estimates of China's CO_2 fluxes.

Inversion	Time range	Observation	Transport model [a]	Prior biosphere flux	Prior ocean flux	Fossil fuels
Jena CarboScope [1]	1957-2022	Ground-based	TM3 (3.85°×5°)	zero	CarboScope v2023	GridFED v2023.1
Copernicus Atmosphere Monitoring Service (CAMS) [2]	1979-2022	Ground-based	LMDz v6 (2.5°×1.27°)	ORCHIDEE and GFEDv4. 1s	CMEMSLSCEF FNN 2022	
CarbonTracker Europe (CTE) [3]	2001-2022	Ground-based	TM5 (Global 3°×2°, Europe 1°×1°, North America 1°×1°)	SiB4 and GFAS	CarboScope v2022	
NISMON [4]	1990-2022	Ground-based	NICAM-TM (~223 km)	VISIT and GFEDv4. 1s	JMA global ocean mapping	
CT-NOAA [5]	2000-2022	Ground-based	TM5 (Global 3°×2°, North America 1°×1°)	GFEDCASA and GFED_C MS	Ocean inversion fluxes, Takahashi pCO2	
UoE [6]	2001-2022	Ground-based	GEOS-Chem (2°×2.5°)	CASA v1.0 and GFED4.0	Takahashi climatology	
IAPCAS [7]	2001-2022	Ground-based	GEOS-Chem (4°×5°)	CASA v1.0 and GFED4.0	Takahashi climatology	
MIROC [8]	2001-2022	Ground-based	MIROC4-ACTM (2.8°×2.8°)	CASA and VISIT	Takahashi climatology	

CMS-Flux [9]	2010-2022	GOSAT and OCO-2	GEOS-Chem (4°×5°)	CARDAMOM	MOM6	
CAMS [10]	2015-2022	OCO-2	LMDz v6 (2.5°×1.27°)	ORCHIDEE and GFEDv4. 1s	CMEMS - LSCEFFNN 2022	
THU [11]	2015-2022	OCO-2	GEOS-Chem (4°×5°)	SiB4.2 and GFEDv4. 1s	Takahashi climatology	
COLA [12]	2015-2022	OCO-2	GEOS-Chem (2°×2.5°)	SiB4 and GFAS	CarboScope v2022	
GCASv2 [13]	2015-2022	OCO-2	MOZART-4 (2.5°×1.875°)	BEPS and GFEDv4. 1	JMA_co2map_v 2023	MEIC v1.4 before 2020, and scaled to match CarbonMonitor afterwards
GONGGA [14]	2015-2022	OCO-2	GEOS-Chem (2°×2.5°)	ORCHIDEE-MICT and GFEDv4. 1	Takahashi Climatology	
GONGGA-CN [15]	2015-2022	OCO-2	WRF-CMAQ (~ 50 km) [16]	ORCHIDEE-MICT and GFEDv4. 1	Takahashi Climatology	

Table S2 Inversion-based estimates of China's CH_4 emissions.

	Time range	Observation	Transport model [a]	Prior anthropogenic fluxes [b]	Prior wetland fluxes [b]	Prior biomass burning fluxes [b]
Bergamaschi 2013 [17]	2000–2010	SCIAMACHY + ground-based	TM5 (4°×5°)	EDGARv4.2	[18]/LPJ WHyMe [19]	GFEDv3.1
Thompson 2015 [20]	2000–2011	Ground-based	FLEXPART	EDGARv4.2 FT2010	[18]	GFEDv3
Deng 2022 [b] [21]	2000–2017	Ground-based or GOSAT	GCP ensemble of 20 inversions Varied transport models	Varied setups for the GCP ensemble. See Table S6 of [22] for details.		
Stavert 2022 [b] [23]	2000–2017	Ground-based or GOSAT	GCP ensemble of 20 inversions Varied transport models			
Wang 2021 [24]	2009–2018	GOSAT + surface	NIES-TM (2.5°×2.5°) + FLEXPART [d]	EDGARv5.0	VISIT	GFASv1.2
Wang 2019 [25]	2010–2012	GOSAT + surface	NIES-TM (2.5°×2.5°) + FLEXPART [d]	EDGARv4.3.2	VISIT	GFASv1.2
Miller 2019 [26]	2010–2015	GOSAT	GEOS-Chem (2°×2.5°)	EDGARv4.2	[27]	GFEDv4
Lu 2021 [28]	2010–2017	GOSAT + surface + ship + aircraft	GEOS-Chem (4°×5°)	EDGAR v4.3.2+GFEI [e]	WetCHARTs 1.2.1	GFEDv4
Sheng 2021 [29]	2010–2017	GOSAT + surface	UK Met Office NAME	EDGAR v4.2/4.3.2 + [30] [e]	WetCHARTS	QFED
Zhang 2022 [31]	2010-2017	GOSAT + surface	GEOS-Chem (0.5°×0.625°)	EDGAR v4.3.2/ PKU_CH4 v2/ EDGAR v5.0/	WetCHARTS	GFEDv4

				CEDS v2021-04-21		
Zhang 2021 [32]	2010–2018	GOSAT	GEOS-Chem (4°×5°)	EDGAR v4.3.2+GFEI [e]	WetCHARTS	GFEDv4s
Janardanan 2020 [33]	2011–2017	GOSAT + surface + ship + aircraft	NIES-TM (2.5°×2.5°) + FLEXPART [c]	EDGAR v4.3.2 [f]	VISIT	GFASv1.2
Zhao 2024 [34]	2011-2021	GOSAT	GEOS-Chem (2°×2.5°)	EDGAR v6.0/ EDGAR v7.0/CTE posterior	WetCHARTS/ CTE posterior	GFEDv4/ CTE posterior
Chen 2022 [35]	2019	TROPOMI	GEOS-Chem (0.25°x0.3125°)	EDGAR v4.3.2+GFEI[e,f]	WetCHARTs v1.3.1	GFEDv4s
Qu 2021 [36]	2019	GOSAT	GEOS-Chem (2°×2.5°)	EDGAR v4.3.2+GFEI [e]	WetCHARTs v1.3.1	GFEDv4s
Liang 2023 [37]	2019	GOSAT/TROPOMI	GEOS-Chem (0.5°×0.625°)	EDGAR v4.3.2+[30][e]	WetCHARTs v1	QFED

[a] Grid resolutions for Euler transport models are listed.

[b] In addition to anthropogenic, wetland, and biomass burning sources, most inversions also include minor natural fluxes such as geological seeps, termites, and soil absorption in their prior fluxes.

[c] Both studies used the inversion ensemble from Saunois et al. (2020). We extract total fluxes from Stavert et al. (2022) and anthropogenic fluxes from Deng et al. (2022).

[d] NIES-TM-FLEXPART is a hybrid modeling system. TM is a Eulerian transport model while FLEXPART is a Lagrangian transport model.

[e] Anthropogenic emissions are from different versions of EDGAR, with fossil fuel emissions replaced by GFEI or with coal mining emissions replaced by Sheng et al. (2019).

Supplementary Figures

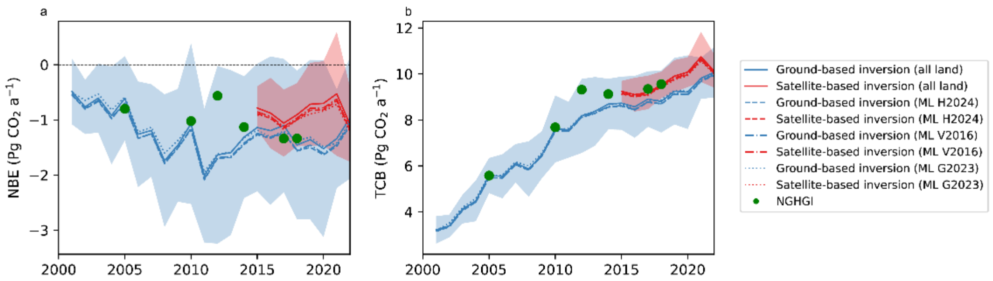

Figure S1. Comparison between inverted NBE and TCB using different maps to represent managed land. The three maps are: 1) H2024 taken from [38], which differentiate managed/secondary and unmanaged/primary forests; 2) V2016 taken from [39], which integrates eight human pressure variables, such as population density, human land use and infrastructure and human access, into a human footprint map. The grid cells with a human footprint value larger than 0 are considered as managed land; and 3) G2023 taken from [40], which defines "intact" forest using remote sensing data.

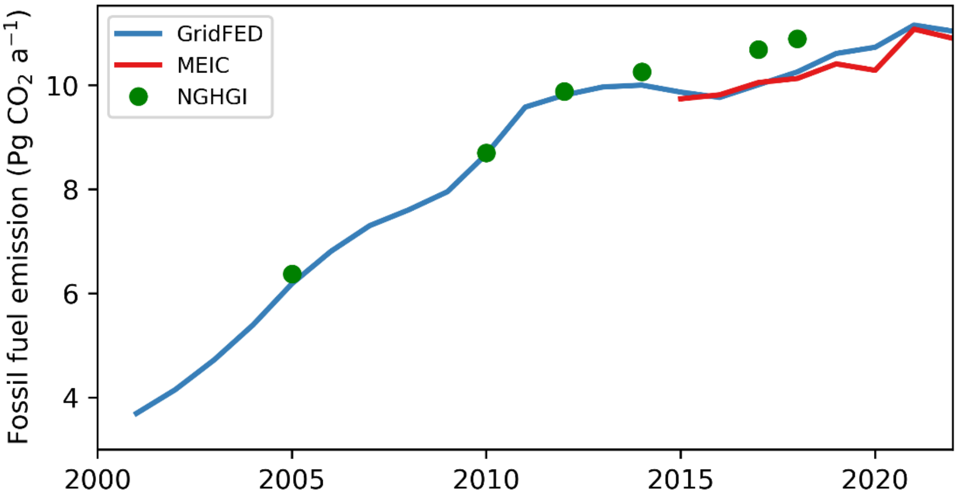

Figure S2. China's fossil fuel and cement CO_2 emissions from GridFED (used in GCP inversions) and MEIC (used in CNGHG inversions), compared to NGHGI.

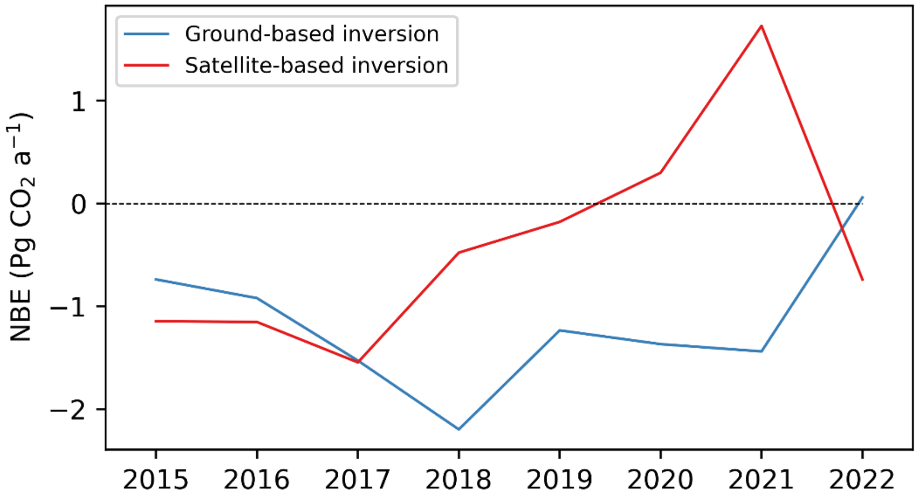

Figure S3. Inverted China's NBE from the CAMS inversion system assimilating ground-based (blue) and satellite (red) observations.

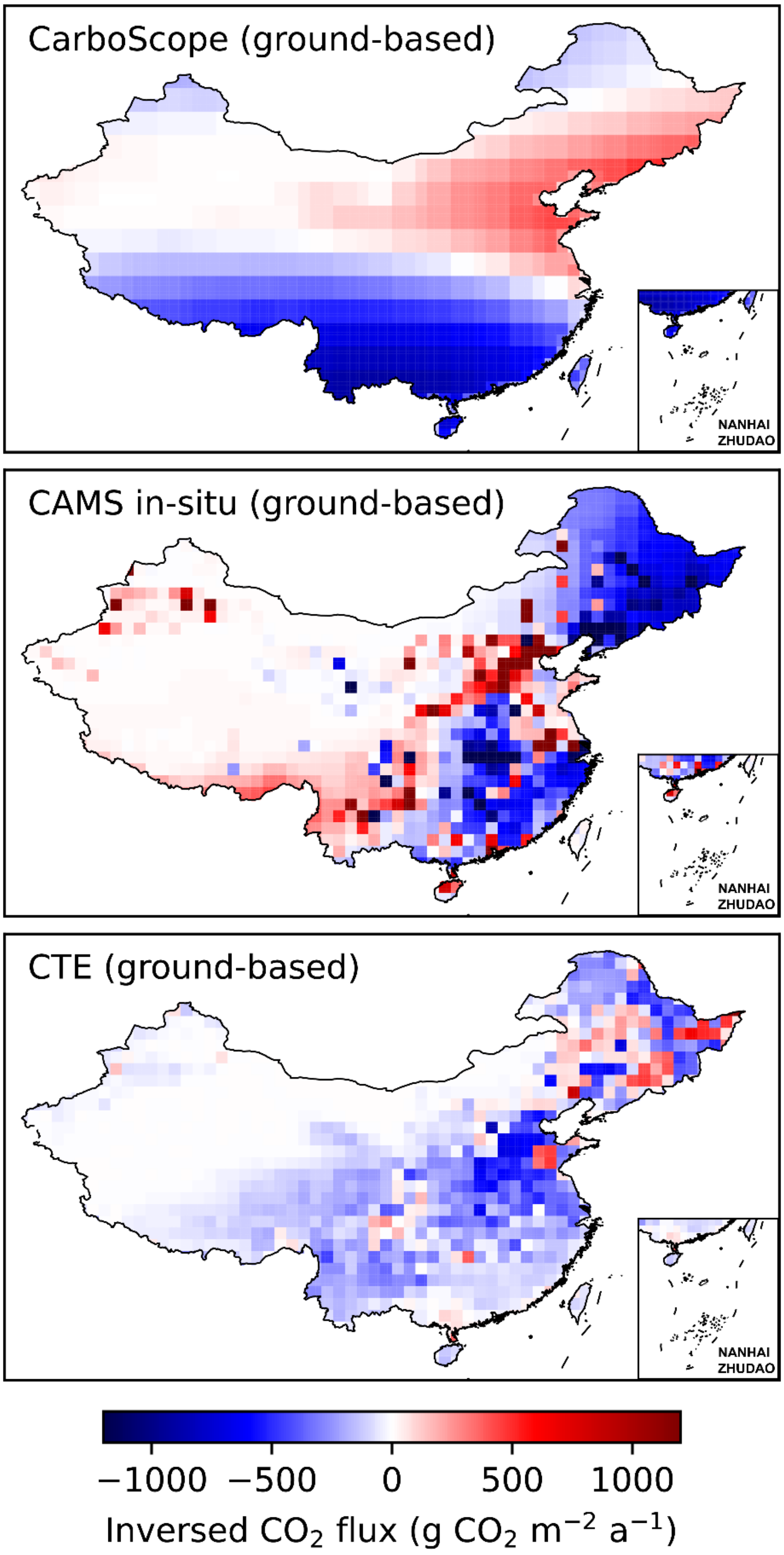

审图号：GS京（2025）0342号

Figure S4. Spatial distribution of annual mean CO_2 fluxes from terrestrial ecosystem in China over the period 2015-2022, during which both ground- and satellite- based inversions are available. The texts in parenthesis indicate which type of observations are assimilated in the inversions.

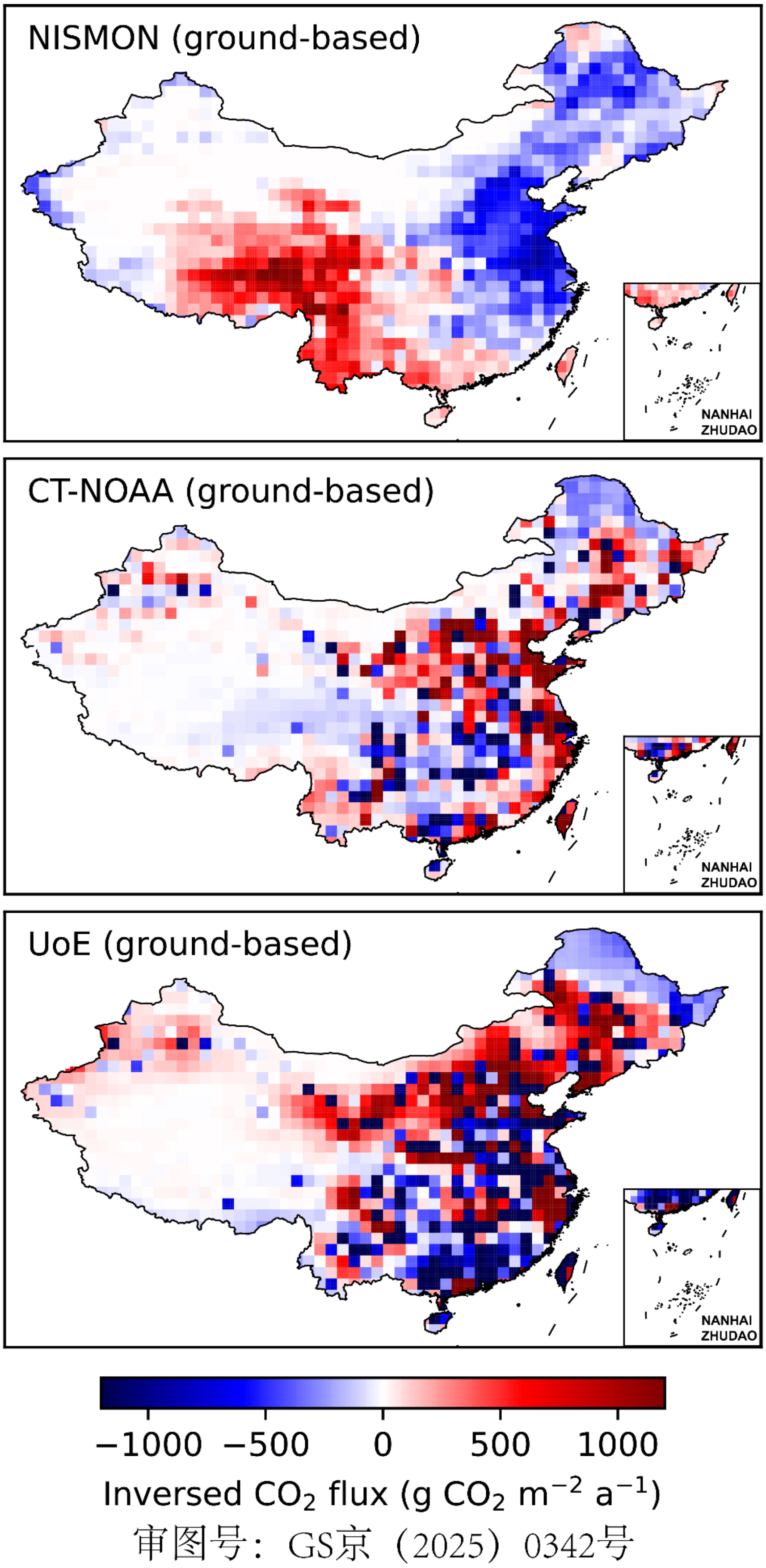

Figure S4. (continued)

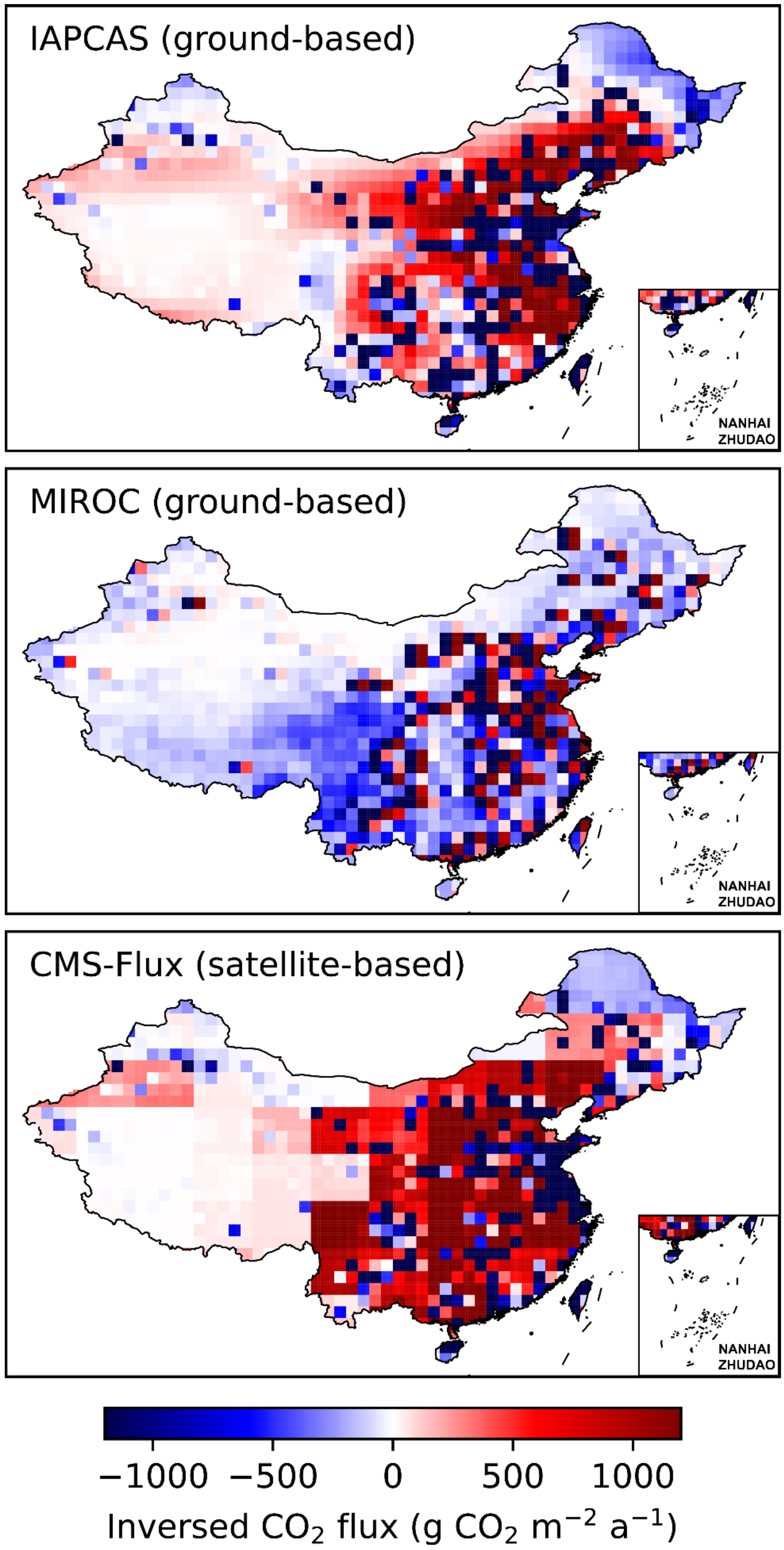

审图号：GS京（2025）0342号

Figure S4. (continued)

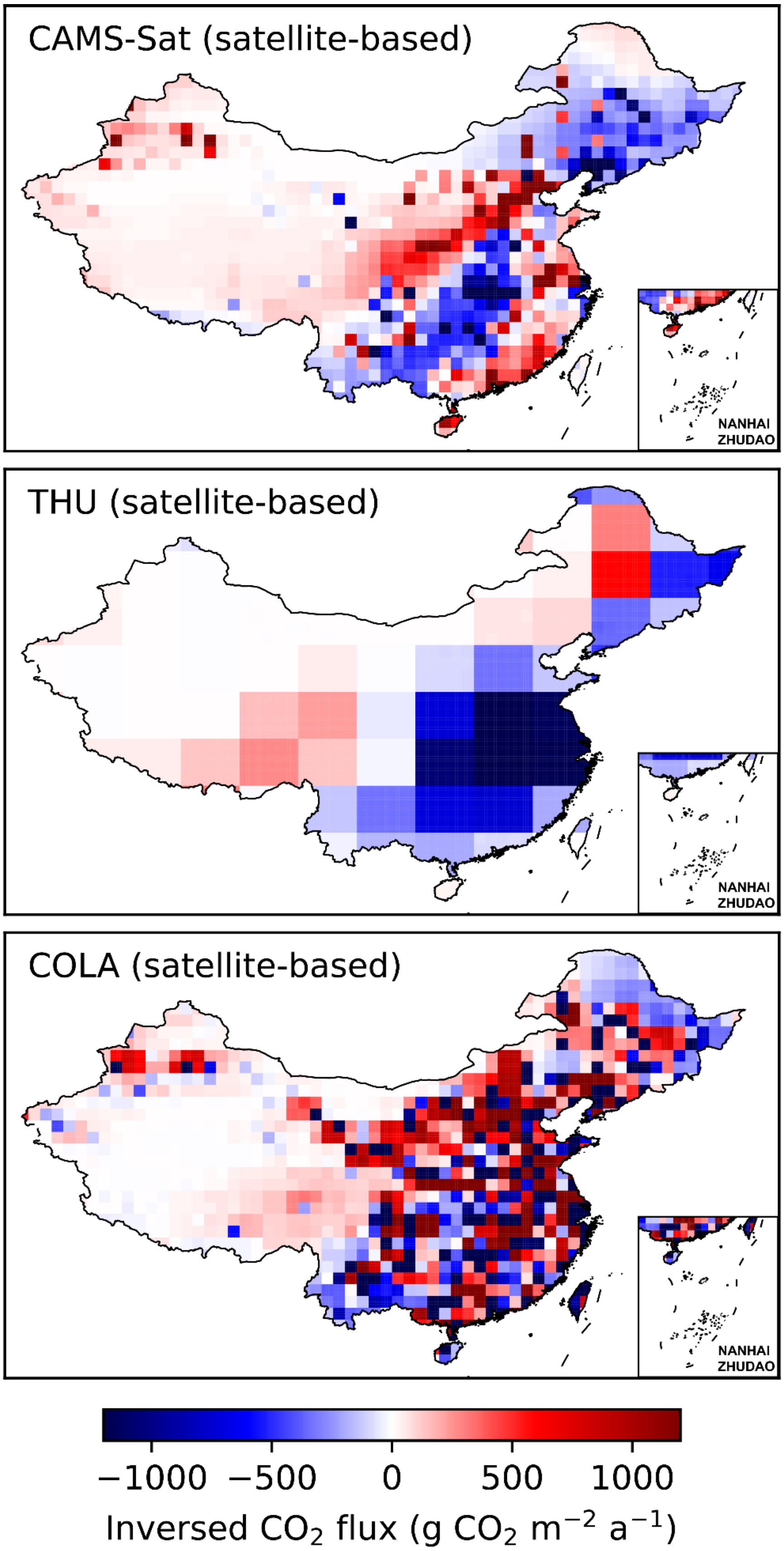

审图号：GS京（2025）0342号

Figure S4. (continued)

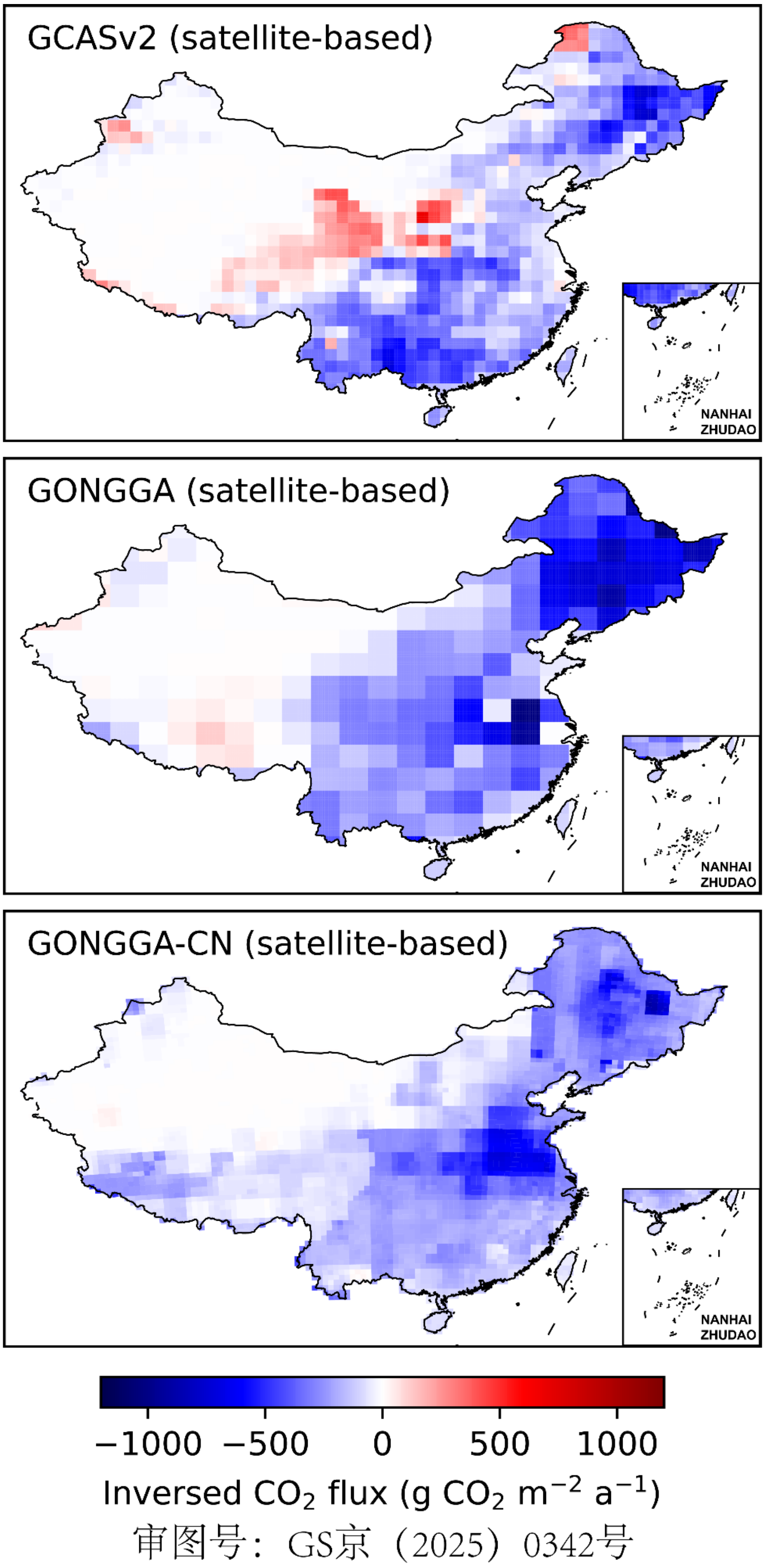

Figure S4. (continued)

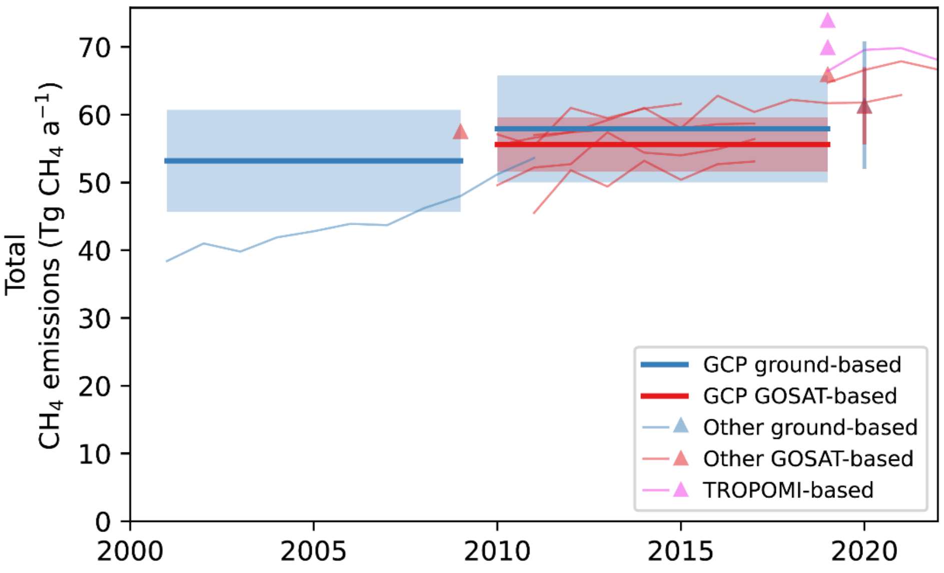

Figure S5. China's total CH_4 emissions from inversions. The thick red and blue lines represent mean values of the GCP ensemble of inversions, and the shaded area represent the 1-σ uncertainty envelope. The light lines and triangles are the estimates from other individual inversions than the GCP ones.

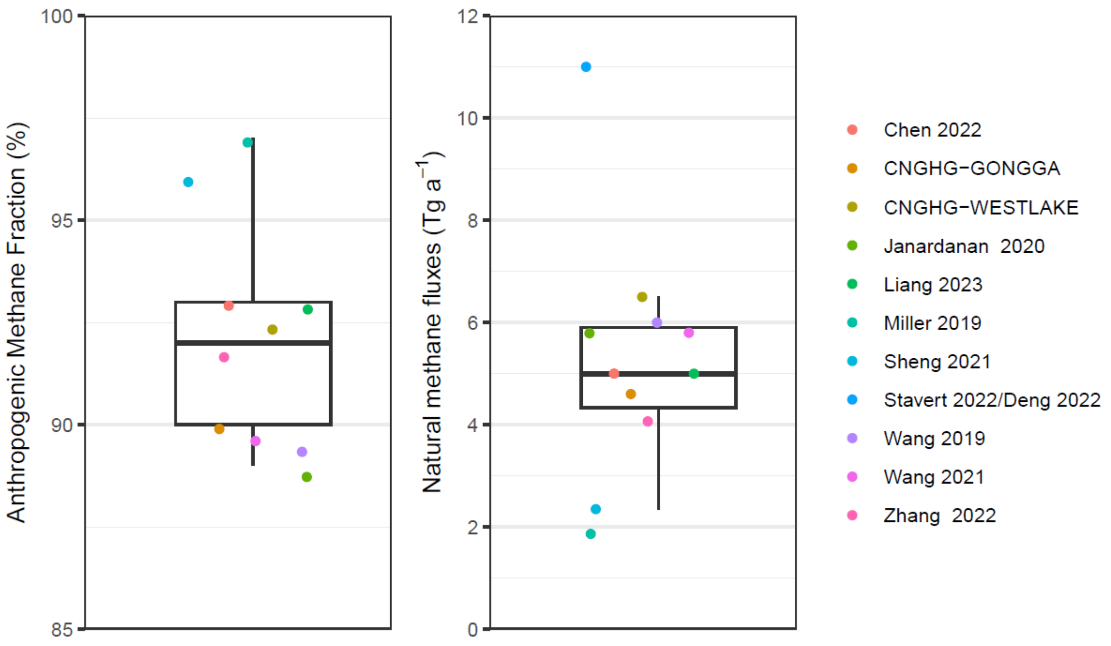

Figure S6. Uncertainties in the partition of anthropogenic and natural methane fluxes. (Left) Fractions of anthropogenic fluxes and (Right) magnitude of natural fluxes reported by different inversion studies.

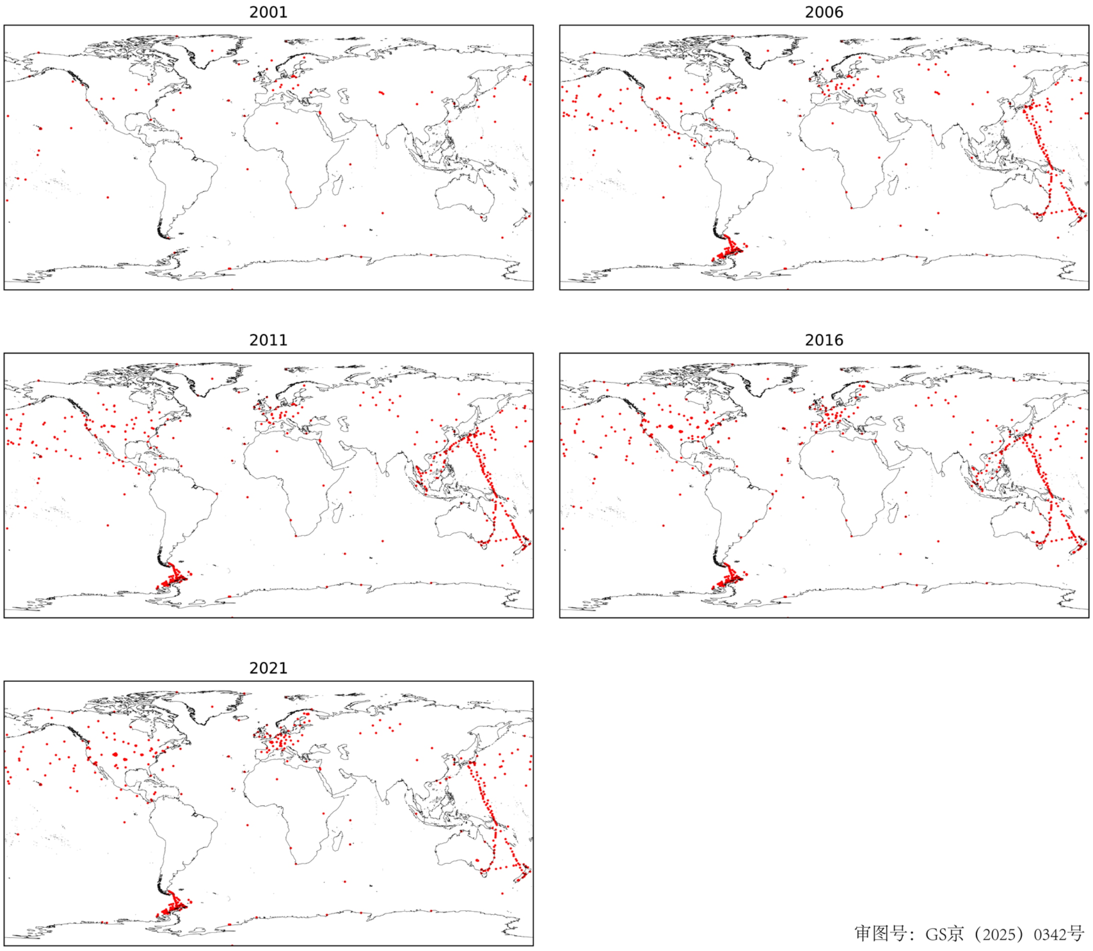

Figure S7. Locations of measurements included in the ObsPack CO_2 dataset with an obs_flag value of 1, indicating measurements with large spatial scale representation. Measurements from shipboard are thinned by a factor of 1000.

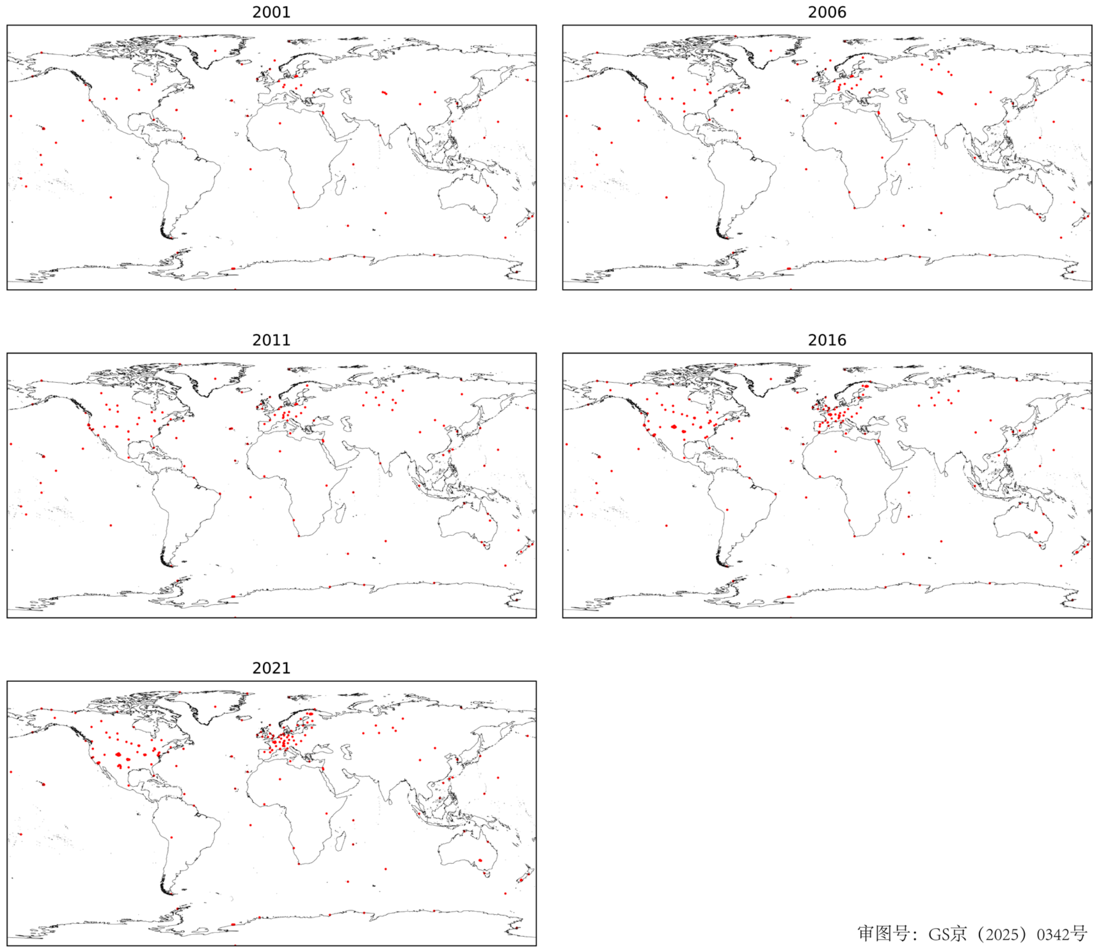

Figure S8. Locations of measurements included in the ObsPack CH_4 dataset with an obs_flag value of 1, indicating measurements with large spatial scale representation. Measurements from shipboard are thinned by a factor of 1000.

References

1. Rödenbeck C, Zaehle S, Keeling R *et al.* How does the terrestrial carbon exchange respond to inter-annual climatic variations? A quantification based on atmospheric CO_2 data. *Biogeosciences* 2018; **15**: 2481–98.

2. Chevallier F, Fisher M, Peylin P *et al.* Inferring CO2 sources and sinks from satellite observations: Method and application to TOVS data. *J Geophys Res* 2005; **110**, DOI: 10.1029/2005jd006390.

3. van der Laan-Luijkx IT, van der Velde IR, van der Veen E *et al.* The CarbonTracker Data Assimilation Shell (CTDAS) v1.0: implementation and global carbon balance 2001–2015. *Geosci Model Dev* 2017; **10**: 2785–800.

4. Niwa Y, Tomita H, Satoh M *et al.* A 4D-Var inversion system based on the icosahedral grid model (NICAM-TM 4D-Var v1.0) – Part 1: Offline forward and adjoint transport models. *Geoscientific Model Development* 2017; **10**: 1157–74.

5. Peters W, Miller JB, Whitaker J *et al.* An ensemble data assimilation system to

estimate CO2 surface fluxes from atmospheric trace gas observations. *J Geophys Res Atmos* 2005; **110**, DOI: 10.1029/2005JD006157.

6. Palmer PI, Feng L, Baker D *et al.* Net carbon emissions from African biosphere dominate pan-tropical atmospheric CO 2 signal. *Nat Commun* 2019; **10**: 1–9.

7. Yang D, Liu Y, Feng L *et al.* The First Global Carbon Dioxide Flux Map Derived from TanSat Measurements. *Adv Atmos Sci* 2021; **38**: 1433–43.

8. Chandra N, Patra PK, Niwa Y *et al.* Estimated regional CO_2 flux and uncertainty based on an ensemble of atmospheric CO_2 inversions. *Atmos Chem Phys* 2022; **22**: 9215–43.

9. Liu J, Baskaran L, Bowman K *et al.* Carbon Monitoring System Flux Net Biosphere Exchange 2020 (CMS-Flux NBE 2020). *Earth Syst Sci Data* 2021; **13**: 299–330.

10. Remaud M, Chevallier F, Cozic A *et al.* On the impact of recent developments of the LMDz atmospheric general circulation model on the simulation of CO_2 transport. *Geosci Model Dev* 2018; **11**: 4489–513.

11. Kong Y, Zheng B, Zhang Q *et al.* Global and regional carbon budget for 2015–2020 inferred from OCO-2 based on an ensemble Kalman filter coupled with GEOS-Chem. *Atmos Chem Phys* 2022; **22**: 10769–88.

12. Liu Z, Zeng N, Liu Y *et al.* Improving the joint estimation of CO_2 and surface carbon fluxes using a constrained ensemble Kalman filter in COLA (v1.0). *Geosci Model Dev* 2022; **15**: 5511–28.

13. Jiang F, Ju W, He W *et al.* A 10-year global monthly averaged terrestrial net ecosystem exchange dataset inferred from the ACOS GOSAT v9 XCO_2 retrievals (GCAS2021). *Earth Syst Sci Data* 2022; **14**: 3013–37.

14. Jin Z, Tian X, Wang Y *et al.* A global surface CO_2 flux dataset (2015–2022) inferred from OCO-2 retrievals using the GONGGA inversion system. *Earth Syst Sci Data* 2024; **16**: 2857–76.

15. Yuan W, Liang M, Gao Y *et al.* The greenhouse gas budget of China during 2000-2023. *In review* 2025.

16. Wang Y, Tian X, Duan M *et al.* Optimal design of surface CO2 observation network to constrain China's land carbon sink. *Sci Bull* 2023; **68**: 1678–86.

17. Bergamaschi P, Houweling S, Segers A *et al.* Atmospheric CH4 in the first decade of the 21st century: Inverse modeling analysis using SCIAMACHY satellite retrievals and NOAA surface measurements. *J Geophys Res Atmos* 2013; **118**: 7350–69.

18. Bergamaschi P, Frankenberg C, Meirink JF *et al.* Satellite chartography of atmospheric methane from SCIAMACHY on board ENVISAT: 2. Evaluation based on inverse model simulations. *J Geophys Res Atmos* 2007; **112**, DOI: 10.1029/2006JD007268.

19. Spahni R, Wania R, Neef L *et al.* Constraining global methane emissions and uptake by ecosystems. *Biogeosciences* 2011; **8**: 1643–65.

20. Thompson RL, Stohl A, Zhou LX *et al.* Methane emissions in East Asia for 2000–2011 estimated using an atmospheric Bayesian inversion. *J Geophys Res Atmos* 2015; **120**: 4352–69.

21. Deng Z, Ciais P, Tzompa-Sosa ZA *et al.* Comparing national greenhouse gas budgets reported in UNFCCC inventories against atmospheric inversions. *Earth Syst Sci Data* 2022; **14**: 1639–75.
22. Saunois M, Stavert AR, Poulter B *et al.* The Global Methane Budget 2000–2017. *Earth Syst Sci Data* 2020; **12**: 1561–623.
23. Stavert AR, Saunois M, Canadell JG *et al.* Regional trends and drivers of the global methane budget. *Glob Chang Biol* 2022; **28**: 182–200.
24. Wang F, Maksyutov S, Janardanan R *et al.* Interannual variability on methane emissions in monsoon Asia derived from GOSAT and surface observations. *Environ Res Lett* 2021; **16**: 024040.
25. Wang F, Maksyutov S, Tsuruta A *et al.* Methane Emission Estimates by the Global High-Resolution Inverse Model Using National Inventories. *Remote Sensing* 2019; **11**: 2489.
26. Miller SM, Michalak AM, Detmers RG *et al.* China's coal mine methane regulations have not curbed growing emissions. *Nat Commun* 2019; **10**: 303.
27. Pickett-Heaps CA, Jacob DJ, Wecht KJ *et al.* Magnitude and seasonality of wetland methane emissions from the Hudson Bay Lowlands (Canada). *Atmos Chem Phys* 2011; **11**: 3773–9.
28. Lu X, Jacob DJ, Zhang Y *et al.* Global methane budget and trend, 2010–2017: complementarity of inverse analyses using in situ (GLOBALVIEWplus CH_4 ObsPack) and satellite (GOSAT) observations. *Atmos Chem Phys* 2021; **21**: 4637–57.
29. Sheng J, Tunnicliffe R, Ganesan AL *et al.* Sustained methane emissions from China after 2012 despite declining coal production and rice-cultivated area. *Environ Res Lett* 2021; **16**: 104018.
30. Sheng J, Song S, Zhang Y *et al.* Bottom-Up Estimates of Coal Mine Methane Emissions in China: A Gridded Inventory, Emission Factors, and Trends. *Environ Sci Technol Lett* 2019; **6**: 473–8.
31. Zhang Y, Fang S, Chen J *et al.* Observed changes in China's methane emissions linked to policy drivers. *Proc Natl Acad Sci USA* 2022; **119**: e2202742119.
32. Zhang Y, Jacob DJ, Lu X *et al.* Attribution of the accelerating increase in atmospheric methane during 2010–2018 by inverse analysis of GOSAT observations. *Atmos Chem Phys* 2021; **21**: 3643–66.
33. Janardanan R, Maksyutov S, Tsuruta A *et al.* Country-Scale Analysis of Methane Emissions with a High-Resolution Inverse Model Using GOSAT and Surface Observations. *Remote Sensing* 2020; **12**: 375.
34. Zhao M, Tian X, Wang Y *et al.* Slowdown in China's methane emission growth. *Natl Sci Rev* 2024; **11**: nwae223.
35. Chen Z, Jacob DJ, Nesser H *et al.* Methane emissions from China: a high-resolution inversion of TROPOMI satellite observations. *Atmos Chem Phys* 2022; **22**: 10809–26.
36. Qu Z, Jacob DJ, Shen L *et al.* Global distribution of methane emissions: a comparative inverse analysis of observations from the TROPOMI and GOSAT satellite instruments. *Atmos Chem Phys* 2021; **21**: 14159–75.

37. Liang R, Zhang Y, Chen W *et al.* East Asian methane emissions inferred from high-resolution inversions of GOSAT and TROPOMI observations: a comparative and evaluative analysis. *Atmos Chem Phys* 2023; **23**: 8039–57.
38. He Y, Piao S, Ciais P *et al.* Future land carbon removals in China consistent with national inventory. *Nat Commun* 2024; **15**: 10426.
39. Venter O, Sanderson EW, Magrach A *et al.* Sixteen years of change in the global terrestrial human footprint and implications for biodiversity conservation. *Nat Commun* 2016; **7**: 12558.
40. Grassi G, Schwingshackl C, Gasser T *et al.* Harmonising the land-use flux estimates of global models and national inventories for 2000–2020. *Earth Syst Sci Data* 2023; **15**: 1093–114.
